# Reproducibility of the Structural Brain Connectome Derived from Diffusion Tensor Imaging

**DOI:** 10.1371/journal.pone.0135247

**Published:** 2015-09-02

**Authors:** Leonardo Bonilha, Ezequiel Gleichgerrcht, Julius Fridriksson, Chris Rorden, Jesse L. Breedlove, Travis Nesland, Walter Paulus, Gunther Helms, Niels K. Focke

**Affiliations:** 1 Department of Neurology, Medical University of South Carolina, Charleston, SC, United States of America; 2 Department of Communication Sciences and Disorders, University of South Carolina, Columbia, SC, United States of America; 3 Department of Psychology, University of South Carolina, Columbia, SC, United States of America; 4 Department of Clinical Neurophysiology, University of Göttingen, Göttingen, Germany; 5 Department of Cognitive Neurology, MR-Research, University of Göttingen, Göttingen, Germany; 6 Department of Neurology/Epileptology and Hertie Institute of Clinical Brain Research, University of Tübingen, Tübingen, Germany; Wake Forest School of Medicine, UNITED STATES

## Abstract

**Rationale:**

Disruptions of brain anatomical connectivity are believed to play a central role in several neurological and psychiatric illnesses. The structural brain connectome is typically derived from diffusion tensor imaging (DTI), which may be influenced by methodological factors related to signal processing, MRI scanners and biophysical properties of neuroanatomical regions. In this study, we evaluated how these variables affect the reproducibility of the structural connectome.

**Methods:**

Twenty healthy adults underwent 3 MRI scanning sessions (twice in the same MRI scanner and a third time in a different scanner unit) within a short period of time. The scanning sessions included similar T1 weighted and DTI sequences. Deterministic or probabilistic tractography was performed to assess link weight based on the number of fibers connecting gray matter regions of interest (ROI). Link weight and graph theory network measures were calculated and reproducibility was assessed through intra-class correlation coefficients, assuming each scanning session as a rater.

**Results:**

Connectome reproducibility was higher with data from the same scanner. The probabilistic approach yielded larger reproducibility, while the individual variation in the number of tracked fibers from deterministic tractography was negatively associated with reproducibility. Links connecting larger and anatomically closer ROIs demonstrated higher reproducibility. In general, graph theory measures demonstrated high reproducibility across scanning sessions.

**Discussion:**

Anatomical factors and tractography approaches can influence the reproducibility of the structural connectome and should be factored in the interpretation of future studies. Our results demonstrate that connectome mapping is a largely reproducible technique, particularly as it relates to the geometry of network architecture measured by graph theory methods.

## Introduction

The comprehensive map of structural neural connectivity at a medium and large scale (the brain connectome) can be reconstructed from white matter Magnetic Resonance (MRI) diffusion tensor imaging (DTI) [1]. White matter diffusion pathways determined through DTI tractography are traditionally considered to be the biophysical representation of axonal bundles and their myelin sheet [2, 3]. The number of DTI tractography streamlines between cortical and subcortical gray matter regions of interest (ROIs) can be used as a measure of the magnitude of connection between ROIs [1, 4] and the iterative process of computing the connectivity between all possible ROIs is applied to reconstruct the whole brain structural network [5]. The resulting network can be subsequently assessed based on the several parameters, such as the presence or absence of connections between ROIs, the weight of regional connectivity, and the geometry of connectivity through graph theory measures [4, 6–8].

The reconstruction of the structural connectome involves a multistep process, encompassing the segmentation of gray matter ROIs (from high resolution T1 weighted images) into anatomically-defined ROIs, and the reconstruction of diffusion streamlines through DTI tractography [9]. Once these steps are independently performed, the tractography data and the segmented ROIs are registered into the same spatial orientation (e.g., co-registered into the diffusion image space), and the overall number of streamlines linking ROIs is computed. Normalization steps can be used to avoid bias in the computation of connectivity strength. For example, the connectivity strength can be normalized based on the distance between ROIs, the size of the ROIs and the surface area of the ROI in the gray and white matter transition [1].

The disruption of normal patterns of structural brain connectivity is believed to play a central role in the pathophysiology of several neurological and psychiatric illnesses, such as epilepsy, dementia, movement disorders and schizophrenia [10–13]. Hence, the computation and assessment of the structural connectome composes a growing subfield within neuroimaging, with rapidly expanding applicability. Furthermore, the use of structural networks to investigate disease mechanisms is constantly improved by advancements in MRI sequence engineering and by increased computational power, enabling the iterative assessment of complex networks.

It is important to recognize that, in spite of several methodological advancements, DTI tractography is still limited by methodological variables. Specifically, tractography can be influenced by MRI signal to noise ratio, voxel size, number of encoding diffusion directions and magnetic field strength [14]. The reproducibility of tractography can be, in consequence, directly influenced by the reliability of DTI. Also DTI is limited in its ability to resolve the complex anatomy of axonal bundles that can cross, converge and diverge within a single voxel. Hence, the anatomical location of white matter tracts may also play a role in network reproducibility.

We hypothesized that, even though the structural brain connectivity is unlikely to change significantly within a short period of time, the structural connectome measured from DTI may exhibit measurement variations that are related to method reproducibility. We postulated that the reproducibility of connectome is influenced by the approach used to calculate tractography and by the anatomical location of white matter fibers.

We also hypothesized that, while regional variability may occur across different scanning sessions, the overall conformation of network architecture, as measured through graph theory variables, is less likely to vary significantly between sessions and may thus be fairly reproducible.

We assessed the reproducibility of the individual structural connectome from a cohort of healthy subjects who were serially assessed through modern DTI tractography connectivity. In a short period of time, subjects were scanned twice in the same MRI scanner and in a third time in another MRI scanner of the same manufacturer and model. We assessed how different scanners, different tractography approaches and anatomical variables affect connectome reproducibility.

## Methods

### Subjects

We studied 20 subjects recruited from the local community (mean age 34.6 ±10.66 years) with no history of neurological or psychiatric illnesses. All subjects signed an informed consent to participate in this study. The Institutional Review Board at the University of Göttingen approved this study.

### Image acquisition

All subjects underwent a first MRI in a 3T Siemens Magnetom TIM Trio (Siemens Healthcare, Erlangen, Germany) equipped with an 8-channel head coil for signal reception (Invivo, Gainesville, Florida) located at the University of Göttingen, yielding: 1) two 2 MP-RAGE scans with IPAT 2 and averaged after linear co-registration (3D MP-RAGE, TR = 2250ms, TE = 3.2ms, 256×256matrix, 256×256mm FOV, parallel imaging GRAPPA = 2); and 2) diffusion single-shot EPI scan (30-directions with b = 1000 s/mm^2^, TR = 10000ms, TE = 93ms, 128×128 matrix parallel imaging GRAPPA = 2, FOV = 243x243 mm, isotropic 1.9 mm voxel size).

All subjects underwent a second scanning session in the same scanner yielding similar images. The average time between Time 1 and Time 2 in scanner A was 126.4 ±102.8 days (range 12–442).

Finally, all subjects underwent a third scanning session, this time in a different physical unit of the same type of MRI scanner (3T Siemens TIM Trio), equipped with a different head coil (12-channel) employing the same imaging sequences. This scanner was also located at the University of Göttingen. The average time between Time 1 Scanner A and Time 1 in scanner B was 158.4 ±103.6 days (range 21–465).

### Image processing

All DICOM images were converted to NIfTI format, and diffusion gradients were extracted using the software mriconvert (http://lcni.uoregon.edu/~jolinda/MRIConvert). The package FMRIB Software Library (FSL)’s Diffusion Toolkit (FDT) was utilized for preprocessing of diffusion-weighted images (DWIs) and for diffusion tensor estimation[15]. The DWIs underwent eddy current correction through affine transformation of each DWI to the base b = 0 s/mm^2^ T2-weighted image.

Gray matter ROIs were obtained from an automatic cortical and subcortical segmentation of the T1-weighted images employing FreeSurfer [16, 17] (http://surfer.nmr.mgh.harvard.edu/) with parcellation into anatomical ROIs according to the Lausanne anatomical atlas, distributed as part of the Connectome Mapping Toolkit (http://www.connectome.ch), yielding 83 ROIs in the subjects’ native T1-weighted space (a total of 42 regions with all regions existing in both hemispheres except for brainstem (1 ROI). Essentially 41 regions are in both hemispheres.) (S1 Table). The ROIS were transformed from the native T1 space into each subject’s DTI space using an affine transformation obtained with FSL’s FLIRT.

### Connectivity matrices

For each patient, we performed an estimation of brain connectivity through two commonly used approaches: deterministic tractography and probabilistic tractography. These methods harness advancements from popular and robust neuroimaging tools commonly used by the scientific community.

Probabilistic tractography was estimated by applying FDT’s probabilistic method of fiber tracking [18]. FDT’s BEDPOST was used to build default distributions of diffusion parameters at each voxel, and probabilistic tractography was obtained using FDT’s probtrackx with default parameters, namely 5000 individual pathways drawn through the probability distributions on principle fiber direction, curvature threshold set at 0.2, 200 maximum steps, step length 0.5mm and distance correction (default settings). The connectivity between ROIs was defined as the number of streamlines arriving in one ROI when another ROI was seeded and vice-versa. Seeding and streamline counting was performed in the voxels within the ROI that were located in the boundary between gray and white matter (white matter surface of Freesurfer segmentation). Specifically, the weighted connectivity between ROIs i and j was defined as the number of probabilistic streamlines arriving at the boundary between j and white matter when the voxels in the boundary between i and white matter were seeded, averaged with the number of probabilistic streamlines obtained from the reverse process, i.e, seeding from j boundary, arriving at the I boundary. The calculation of the probabilistic streamlines was corrected based on the distance travelled by the streamline connecting i and j (“distance correction” built into probtrackX). A connectivity matrix A was defined for each subject, where the weighted link Aij corresponded to the number of streamlines connecting i and j divided by the sum of the surface of areas of ROIs i and j.

Deterministic tractography was reconstructed with the software Diffusion Toolkit[19] by seeding all brain voxels. Two types of threshold were used as stopping criterion for fiber tracking. The first one was image mask, i.e., a threshold based on the distribution of the voxel-wise signal intensity observed in the subject’s b0 image, acquired with all diffusion gradients turned off, not sensitive to diffusion direction. This process was performed using the automatic calculation of threshold built into Diffusion Toolkit. The second stopping criterion was the angle threshold, whereby streamlines encountering an angle greater than 45 degrees were stopped. These are default settings.

As expected, the average number of streamlines tracked per subject was lower with deterministic tractography (3.92 x10^5^) compared with probabilistic tractography (4.65 x10^11^). The process of calculation of ROI connectivity from deterministic tractography is different than the process of probabilistic tractography. With probabilistic tractography, different ROIs are seeded and the number of streamlines arriving at other ROIs is counted. With deterministic tractography, seeding occurs across all white matter voxels and the resulting fibers are then assessed one by one, to evaluate whether the fiber extreme-points connect different ROIs. It is possible that some fibers do not connect ROIs. Thus, the weighted connectivity between ROIs i and j was calculated by the number of deterministic fibers with one extreme in the boundary between ROI i and white matter, and the other extreme in the boundary between ROI j and white matter, or vice versa. The connectivity matrix was normalized based on the distance travelled by each fiber connecting i and j, and the surface of areas of ROIs i and j, according to the formula, proposed by Hagmann et al [1]: w(e) = (2/(Si + Sj)) ∑l/l(f), where Si and Sj represent the surface areas of ROIs i and j, l represents the fiber linking i and j, and l(f) is the length travelled by l.

Importantly, the seed ROIs used in the construction of probabilistic connectivity matrices were the same seed ROIs used in the construction of deterministic tractography matrices. The ROIs were obtained from segmentation of the T1 weighted images, which were acquired in the same session as the DTI images were acquired.

S1 Fig provides an overview of the pre-processing steps, demonstrating, for one representative subject, native T1 images with anatomical ROIs, the non-diffusion images, deterministic tractography streamlines and connectivity matrices.

All raw connectome matrices evaluated in this study are available as S1 Data.

### Network properties based on graph theory

For each subject’s connectivity matrix, we calculated graph theory (GT) measures yielding the following parameters: nodal degree (the number of links connecting the node); nodal betweenness centrality (the fraction of all possible network links that involve that node), nodal clustering coefficient (the number of nodes that are neighbors to that node, which are also neighbors of each other); and nodal strength (degree multiplied by the weight of each connection). All GT measures were calculated from non-directed weighted matrices using the brain connectivity toolbox (https://sites.google.com/site/bctnet/).

### Reproducibility

We assessed the reproducibility of connectome mapping by evaluating the intra-class correlation coefficient (ICC) between different time point measurements. The ICC was calculated for the absolute agreement between measurements[20]. Assuming that each MRI session is a different “rater”, we evaluated the ICC for each link across all subjects across different MRI scanning sessions. For example, the connectivity between ROIs i and j was assessed for each subject. Hence, for the link i and j, 20 measurements were obtained for each scanning session, and the ICC was calculated across scanning sessions. We assessed the ICC within the same scanner in two different time points and across different scanners. The ICC was calculated using in-house developed scripts based on the methods “Intraclass Correlation Coefficient (ICC)” by Arash Salarian (https://www.mathworks.com/matlabcentral/fileexchange/22099) for Matlab.

We evaluated whether link-wise reproducibility was influenced by distance between ROIs, the overall volume of ROIs and the ratio between ROI volumes. These relationships were assessed through correlations between link-wise ICCs and anatomical features (totaling 12 correlations, 6 for deterministic tractography measures, with 3 for images from the same scanner and 3 for images from different scanners, and 6 for probabilistic tractography measures).

To investigate if links with high reproducibility from deterministic tractography also demonstrated high reproducibility with probabilistic tractography, we performed correlations between measures (2 correlations in total).

To evaluate whether probabilistic tractography yielded higher link-wise ICCs compared with deterministic tractography, we assessed differences in distribution of ICCs through 2 t-tests (one comparing one method versus another using images from the same scanner, and another comparing methods with images from different scanners). We also evaluated whether links with a higher number of tracked fibers across individuals yielded a higher ICC, and whether links with a higher variability in number across subjects (i.e., the standard deviation divided by mean) was associated with a lower ICC. This was assessed through multiple correlations (4 in total per tractography method).

Finally, we evaluated the reliability of GT measures by investigating the ICC across nodal GT measurements. For each subject, a GT measure was obtained for each ROI (for example, degree for ROI i). Similarly, assuming that each scanning session is a “rater”, the ICC was calculated for each node, for each GT measure, and compared across different time points. This step encompassed 14 correlation analyses

Overall, the level of statistical significance was corrected based on the total number of multiple comparisons described above (38 = 12+2+2+8+14). A Bonferroni adjusted p = 0.0013 (i.e., 0.05/38) was defined as the threshold for statistical significance.

## Results

**Fig 1:**
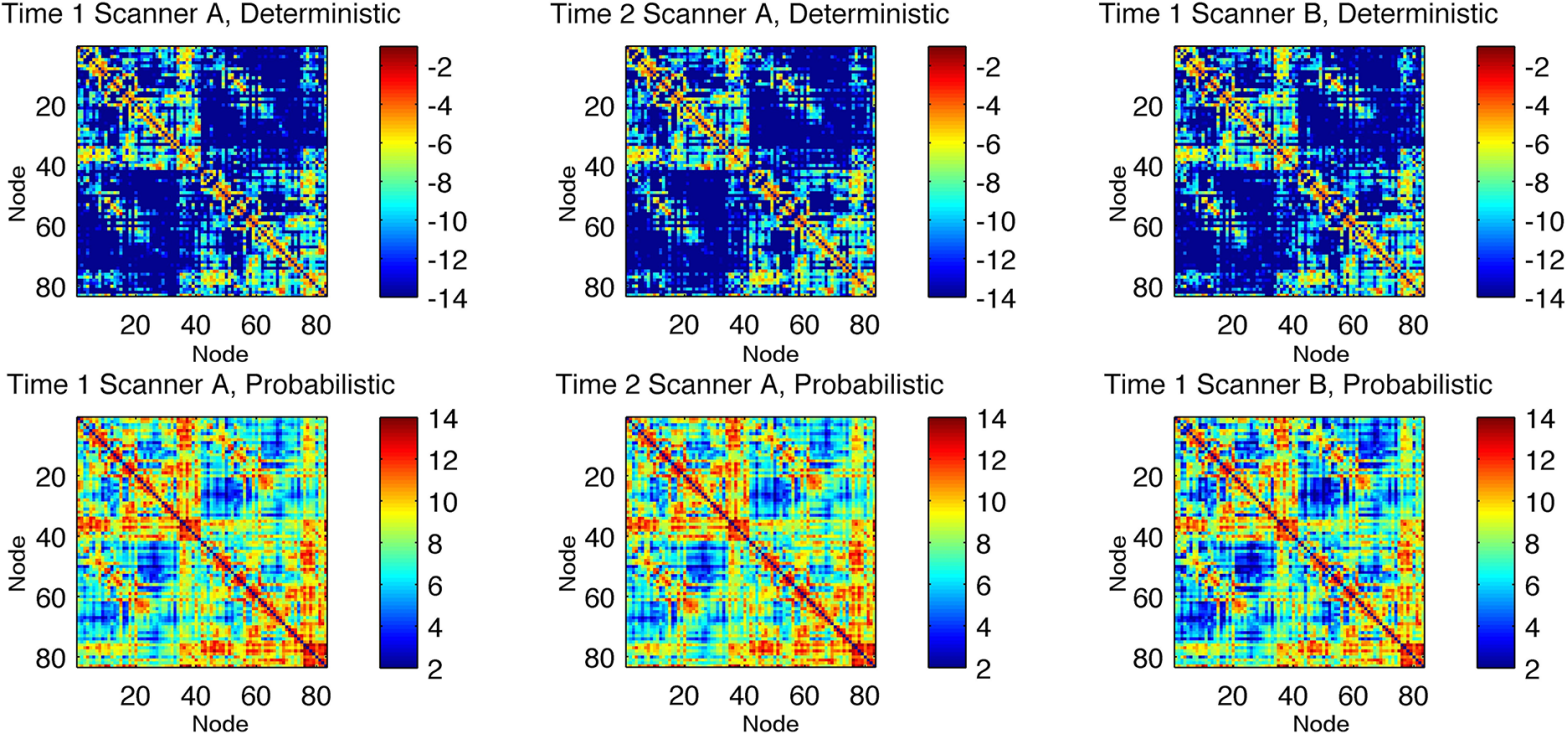
Average connectivity matrices from all subjects for each scanning session (Time 1 and 2 in Scanner A represent measures from the same MRI scanner in different time points, and Time 1 in Scanner B represents a third measure from a different MRI scanner). Each matrix element represents the weighted connectivity between the ROIs indicated by the column and by the row. The color bars indicate log(weighted connectivity).

The average connectivity matrix for each time point and for each tractography modality is displayed in Fig 1. Connectivity matrices generated with deterministic tractography were more sparsely populated compared with connectivity matrices from probabilistic tractography.

**Fig 2:**
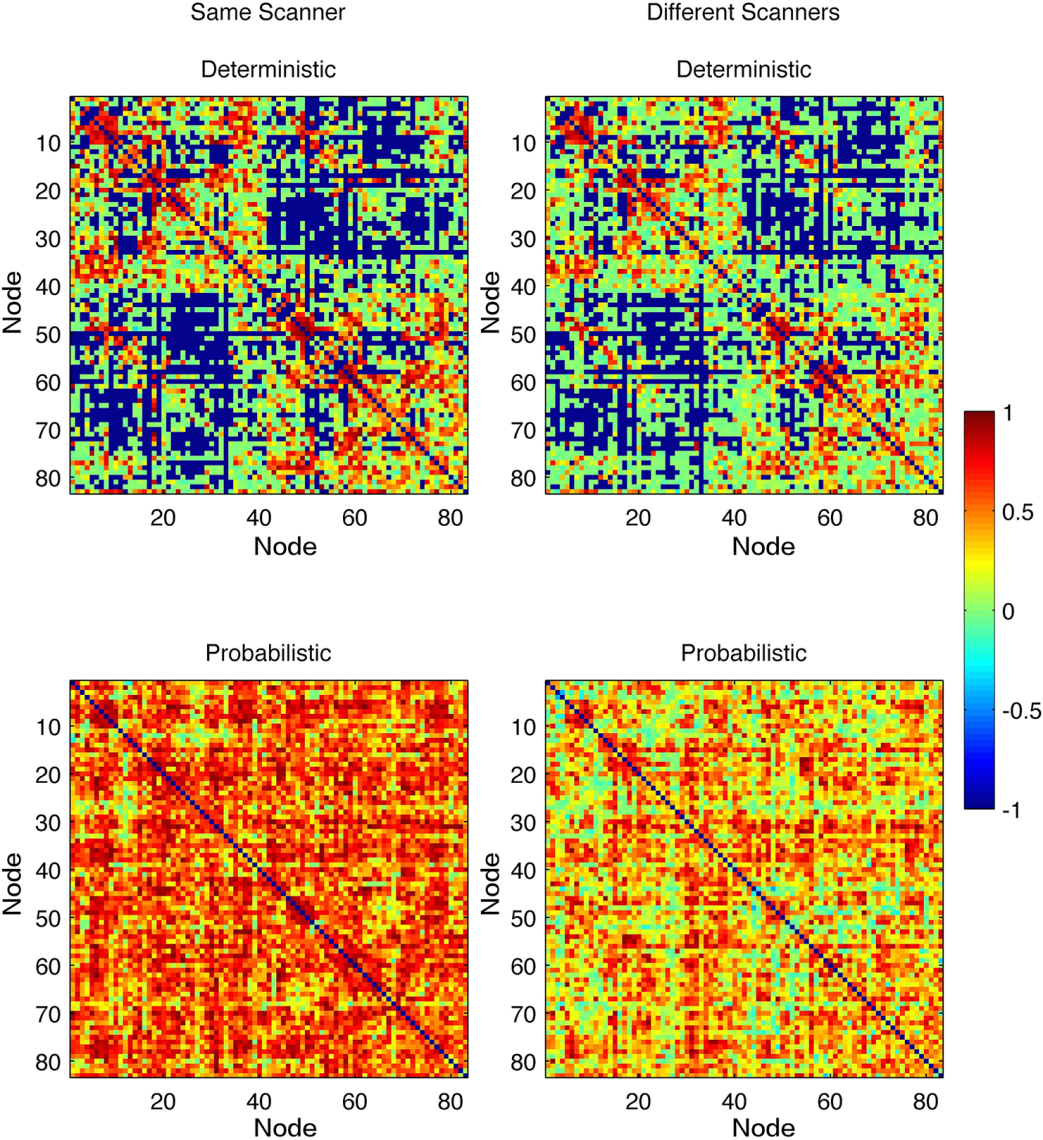
Link-wise ICCs. Each matrix entry represents the ICC observed for the white matter link between the gray matter ROI in the row and the gray matter ROI in the column.

Link-wise ICCs are displayed in Fig 2. ICCs were overall lower when data from two different scanners were assessed compared to data from the same scanner. The histograms demonstrating the distribution of ICCs can be observed in S2 Fig. Higher ICCs were observed with data from the same scanner data compared with different scanners, when employing probabilistic methods (t = -44.97, P<0.00001) and deterministic methods (t = -11.67, P<0.00001).

Probabilistic tractography was associated with overall higher ICCs compared with deterministic tractography, both for data from the same scanner (t = -48.93, P<0.00001) as well as for data from different scanners (t = -27.77, p<0.00001).

**Fig 3:**
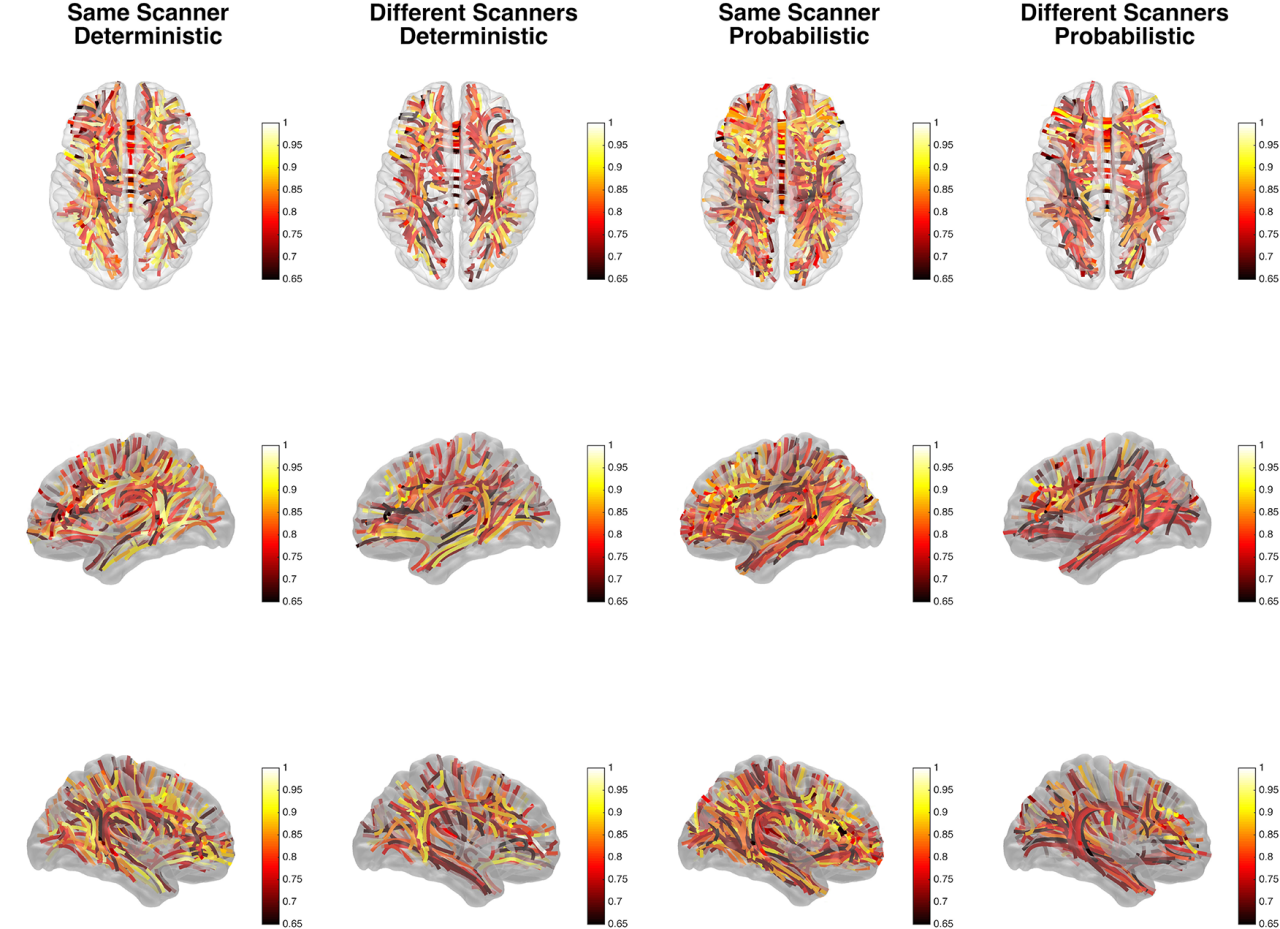
This figure demonstrates each connectome link represented by a line corresponding to the center of mass of the bundle of fibers associated with that link (estimated from deterministic tractography). Each link is color-coded based on its reproducibility per tractography approach and scanner usage. The colorbars indicate the link-wise ICC.

The anatomical location of link-wise ICCs can be observed in Fig 3, which demonstrates each connectome link represented by a line corresponding to the center of mass of the bundle composed by the fibers included in that link (obtained from deterministic tractography). Each link is in turn color-coded based on its reproducibility per tractography approach and scanner usage. As the figure demonstrates, probabilistic tractography and scanning sessions within the same scanner were associated with a higher number of reproducible links and higher ICCs. In fact, probabilistic tractography was associated with a higher number of links with ICC>0.75 compared with deterministic tractography for images obtained from the same scanner (p<0.0001) and from images obtained from different scanners (p<0.0001). S1 Table provides a comprehensive report of link-wise ICCs and the corresponding white matter areas of tractography streamlines traveled per link.

**Fig 4:**
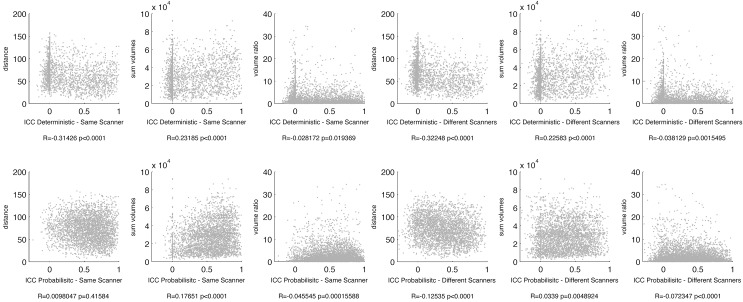
This figure demonstrates the association between the anatomical properties of each link and link-wise reproducibility. The anatomical properties are Euclidian distance between connected gray matter ROIs, sum of the volume of the connected ROIs and the ratio of the volumes between the connected ROIs. Link-wise reproducibility is determined by ICCs. The scatter plots demonstrate each anatomical property in the y-axis, and the ICCs in the x-axis. The statistical relationship between these measures is defined by a correlation coefficient, whose details are displayed below each scatter plot.

We observed significant relationships between link-wise ICC and the structural properties of the connected ROIs. For deterministic tractography, ICCs were lower when the Euclidian distance between ROIs was higher, or when one ROI was notably larger in volume compared with other ROI. ICCs were higher when the sum of the volumes of the connected ROIs was higher. For probabilistic tractography, the same relationships were observed when data from different scanners were evaluated. However, there was not a correlation between distance and ICCs when data from the same scanner were evaluated. These results are summarized in Fig 4.

Links with a high ICC from deterministic tractography were more likely to exhibit a high ICC with probabilistic tractography. S3 Fig demonstrates the significant positive correlations between ICCs from probabilistic tractography and ICCs from deterministic tractography. This relationship was observed with data from the same scanner and with data from different scanners.

For deterministic tractography, there was a positive association between the average number of fibers tracked per link and the link’s ICC (same and different scanners). These data are demonstrated in S4 Fig. Probabilistic tractography did not demonstrate a relationship between ICCs and the number of tracked fibers. However, the dispersion of link weights (i.e., the amount of variability in the number of tracked fibers per link) was negatively associated with ICCs in both deterministic and probabilistic tractography (the latter, only when the data from the same scanner was evaluated). These results are demonstrated in S5 Fig.

**Fig 5:**
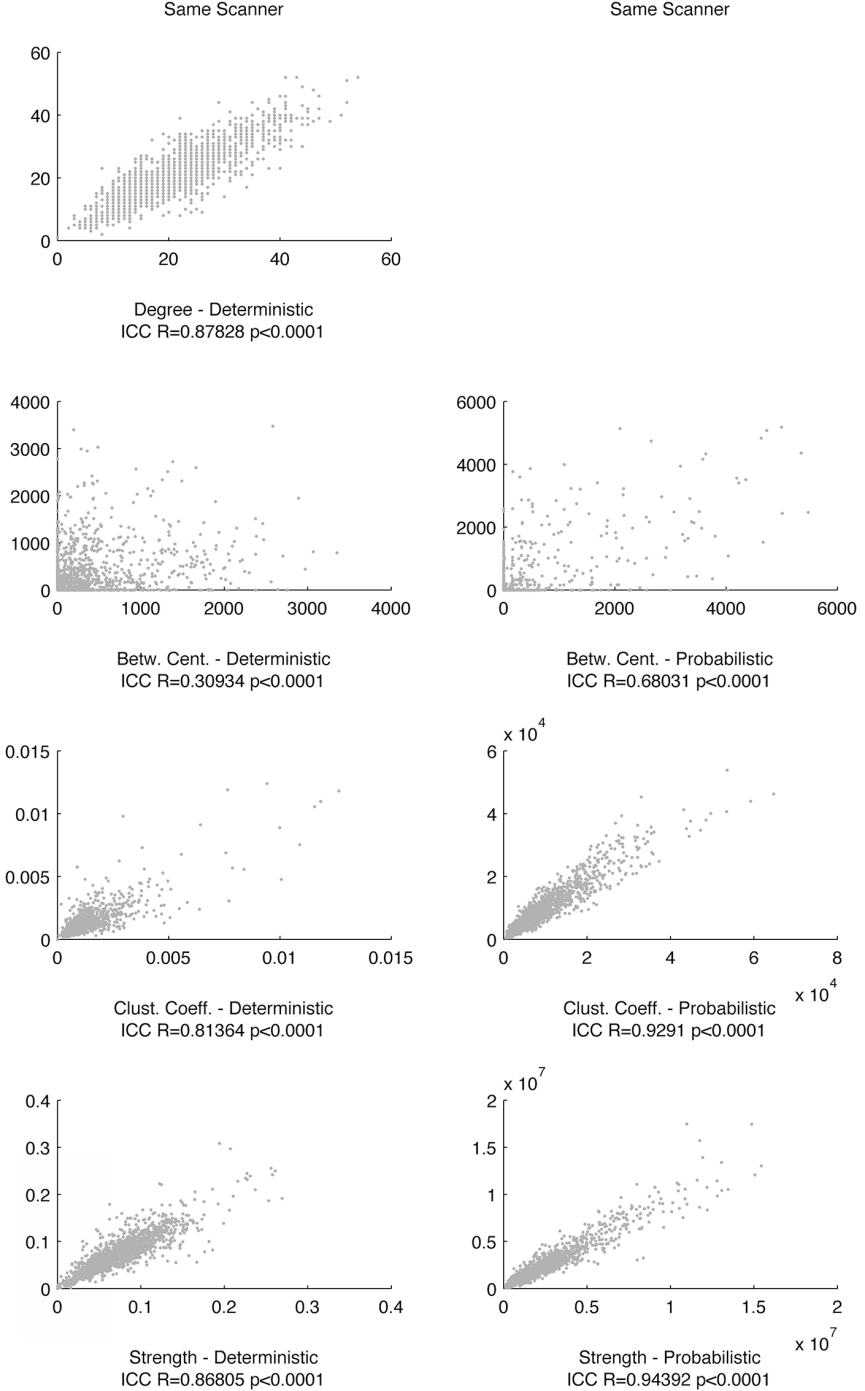
The scatter plots demonstrate the relationship between link-wise graph theory metrics obtained from connectomes calculated from scanning session in time 1 (x-axis) and in time 2 (y-axis) within the same MRI scanner. The scale set for the x-axis is the same as for the y-axis for all graphs. The ICC between each pair of measurements is displayed below each scatter plot. Of note, the relationship between degrees was not assessed for probabilistic tractography given the low sparsity of networks generated from probabilistic methods, therefore leading to a ceiling degree effect.

**Fig 6:**
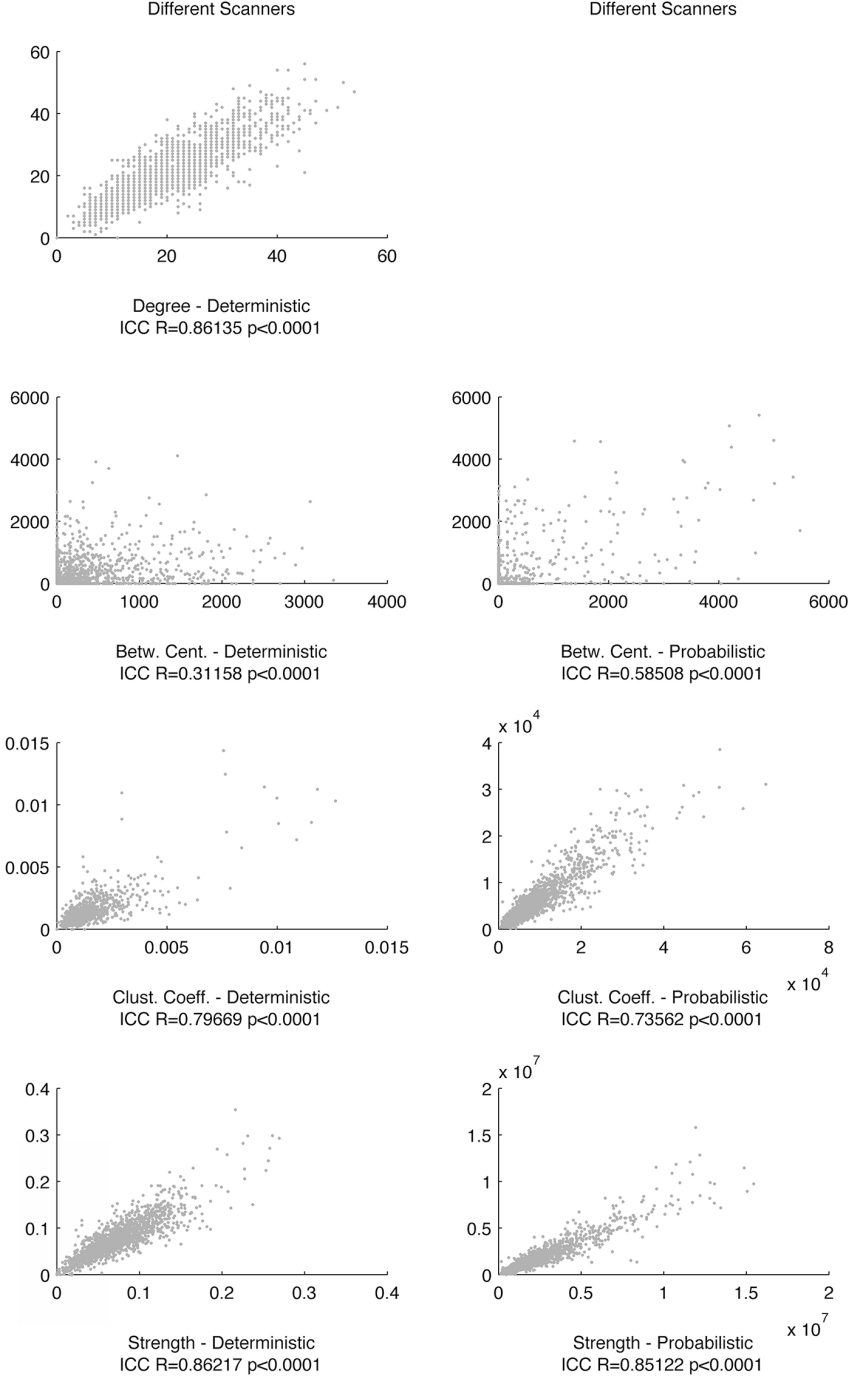
The scatter plots demonstrate the relationship between link-wise graph theory metrics across different scanners (Time 1, Scanner in x-axis and Time 1 Scanner B in y-axis). The ICC between each pair of measurements is displayed below each scatter plot. Similarly, the relationship between degrees was not assessed for probabilistic tractography given the low sparsity of networks generated from probabilistic methods, therefore leading to a ceiling degree effect.

Finally, we observed that graph theory metrics were highly reproducible across different time points in the same scanner and in different scanners for both probabilistic tractography and deterministic tractography. Nodal degree, betweenness centrality, clustering coefficient and strength were significantly correlated across scanning sessions for both tractography modalities. These results are summarized in Figs 5 and 6.

## Discussion

In this study, we evaluated the reproducibility of the whole brain structural connectome derived from DTI. We assessed how reproducibility across time was affected by 1) MRI scanner (i.e., scanning sessions within the same scanner and across different scanners), 2) method of calculating connectivity from DTI fiber tracking, and 3) anatomical properties of each link.

We evaluated images from a cohort of healthy subjects scanned and re-scanned within a relatively short period of time to minimize any real biological variations in structural connectivity. Our main findings are summarized below.

### MRI scanner effect

We observed a higher reproducibility of connectome mapping when subjects were re-scanned in the same scanner. A larger number of links exhibited a higher ICC when comparing data obtained from the same scanner. Nonetheless, links within large white matter pathways, such as the corpus callosum, superior and inferior longitudinal bundles maintained a high ICC within and across scanners.

### Method of calculating connectivity

Probabilistic tractography was associated with a higher number of links with a higher ICC. However it is important to note that for some specific links (as described in S1 Table), the ICC obtained from both tractography methods was equivalent. There was a significant correlation between link-wise ICC from both methodologies, suggesting a consistency in anatomical reproducibility from both tractography approaches.

Deterministic tractography lead to more sparse connectivity matrices (i.e., links with weight equal to, or close to zero). This leads to the speculation that the overall higher reproducibility of probabilistic tractography may be due to those links that are not consistently tracked with deterministic tractography, but resolved with probabilistic methods. Indeed, deterministic tractography reproducibility was also associated with link-weight dispersion (i.e., the variability in the number of fibers per individual). Links with higher inter-subject variability in number of fibers were associated with a lower ICC. This observation suggests that tracks that are more consistently tracked across subjects are also more reproducible.

Importantly, seeding is inherently different between deterministic and probabilistic tractography and it may influence the levels of reproducibility of each method. The purpose of this manuscript is to illustrate the levels of reproducibility of commonly used approaches to reconstruct the structural connectome, rather than a comparison of DTI methods.

### Anatomical link-wise characteristics

Overall, we observed that measurement reproducibility was higher for fibers connecting ROIs with their centers of mass within a shorter Euclidean distance. Measurement reproducibility was also higher for fibers connecting larger ROIs. These are intuitive observations, since connections between ROIs located distant from each other imply that the connecting link travels through a longer distance, with possible anatomical intricacies of the fiber pathways. Fiber tracking is therefore more likely to be interrupted as function of the number of connecting steps in the tracking process[2]. Similarly, larger ROIs are likely to generate a higher number of fibers and therefore increase the likelihood of successful tracking. Interestingly, reproducibility of structural connectivity as a function of the size of the ROIs was maintained even for when fiber tracking was corrected based on the surface or volume of the connecting ROIs.

### Graph theory metrics

Interestingly, we observed a fairly high ICC for nodal graph theory measures when assessing different scanning sessions (both within and across different scanners) for both tractography approaches. These results suggest that the conformation of the neural network remains fairly stable across scanning sessions, in spite of regional variability in fiber tracking. Specifically, the nodal influence on the network, in relationship with the geometry of the nodal connectivity to the network, as statistically equivalent reproducible and equivalent between measurements. These results are encouraging for future connectivity studies employing graph theory based approaches, as they suggest that the overall neural architecture was largely consistent across scanning sessions and methodological approaches.

### General discussion

Given that most neuroimaging studies will employ the same MRI scanner, we believe that our findings related to the reproducibility of data from the same scanner may be our most relevant observation. We noted that reproducibility is overall higher with probabilistic tractography, but also significantly high with deterministic tractography for selected links. Our results suggest that probabilistic and deterministic tractography yield reproducible observations, but deterministic tractography reproducibility is more restricted to specific tracts.

These observations highlight the importance of taking into account the effect of methodological issues when interpreting structural connectivity data. The structural connectome measurement is subjected to the sensitivity and reproducibility of the methodology employed, and biological findings should be interpreted in this context.

Our results expand on previous observation regarding the reproducibility of DTI MRI. Magnotta et al. [21] assessed the multicenter reliability of diffusion tensor imaging, when interpreting voxel-based scalars of diffusion properties. They noted an intra-subject coefficient of variation less than 1%, while the inter-site coefficient of variation increased to ~1%-3%. These results are in accordance with the results from Vollmar et al. [22], who noted an intra-site ICC = 0.90–0.99 and inter-site ICC = 0.82–0.99 in fractional anisotropy measurements. These studies suggest that scalar measures of DTI are fairly reproducible. Our observations are also consistent with previous studies regarding reproducibility of DTI fiber tracking. Heiervang et al. [23] observed that 12 direction data are sufficient for reproducibly defining the core of large bundles but may be less sensitive to smaller pathways. They also observed that measures of MD and FA long tracts were reproducible, with inter-session coefficient of variation below 5%. The high reproducibility of scalar diffusion metrics was also reported by Besseling et al [24]. The findings from Danielian et al. [25] are also particularly relevant: by assessing specific white matter pathways, they demonstrated that same-scan fiber tracking evaluations showed good geometric alignment and reliable diffusion property measurements.

A recent study by Buchanan et al. [26] evaluating the test-retest reliability of connectome measure demonstrated that within-subject differences were smaller than between-subject differences. Our results are largely consistent with their observations, and we expand their conclusion by demonstrating the influence on reproducibility of regional link-wise anatomical localization, measures from different scanners and graph theoretical measurements of network architecture. Interestingly our results also confirm the recent observations that network geometry are reproducible across scanning sessions, as demonstrated by Duda et al. [27] and by Cheng et al. [28], whilst we further demonstrate that tractography approaches do not significantly disrupt this reproducibility.

Interestingly, in an earlier study, Vaessen et al. demonstrated a high reproducibility of small world metrics obtained from connectomes reconstructed from two different sessions in a small group of subjects (n = 6) [29].

We believe that our observations from this present study provide important additional insight into the reproducibility of structural connectivity for three new innovative aspects. First, we evaluated the reproducibility of whole connectome mapping. The concept of the brain connectome is a promising development in neuroscience since several physiological and pathological processes of the human brain are postulated to arise from architectural organization of neural networks[30–33]. Hence, the brain connectome will become an important tool in the investigation of mechanisms underlying health and disease of the nervous system. The understanding of technical limitations and anatomical location of higher reproducibility can greatly improve the interpretation of the findings from connectome-based studies. Second, we compare different tractography approaches. Probabilistic tractography constitutes a robust expansion of fiber tracking, with the possibility to resolve crossing fibers [18]. Nonetheless, it is a time consuming method. By contrasting the reproducibility of probabilistic versus deterministic tractography, we provide further information to guide the decision of one approach versus another in future studies. Third, we compared within and between scanner measures. Even though most studies using the connectome may opt to use data from one scanner, a superb utilization of the connectome is related to longitudinal studies, where, realistically, issues regarding scanner upgrades or replacements may be common. Moreover, multi-site studies are getting increasingly common to generate large datasets or to systematically study rare diseases. Our results can guide the interpretation of data obtained from single or multiple scanners.

### Study limitations

The results from this study should be interpreted in the context of its limitations. First, we did not directly address the relationship between fiber curvature and reproducibility, including the way affine registration may threaten reproducibility among areas prone to distortion. Furthermore, ROIs were converted from the native T1 space into the diffusion space using a linear transformation. This “within-subject” approach reduces more significant distortions associated with individual variability of sulci and gyri positioning. However, diffusion images are subject to different types of distortions compared with T1 images and the affine registration may be insufficient to completely correct for those.

We also did not address the influence of other forms of fiber tracking, such as diffusional kurtosis tractography [34, 35] and high angular resolution diffusion imaging, including DSI [36]. We believe that the interaction between these methods and fiber curvature, fiber crossing and complex link pathways would be a highly relevant topic for future studies. Also of importance for future studies is the effect of scanner drift over time. It must be noted that the scan/re-scan period was variable across subjects, and that slight changes in image registration could occur due to hardware variability over time, especially for longer time scales. Importantly, routine maintenance of the scanners must ensure reliable registration throughout time. The maximum scan/re-scan in our study was nonetheless similar to other studies looking at the longitudinal reproducibility of tractography (e.g. [25]).

Second, our connectome data did not include cerebellar or intra-brain stem pathways. Resolution of standard DTI is ill suited to resolve the complex brainstem architecture in a sufficient way. Thus, we opted to use a single brainstem region that was fully integrated into the reconstruction process allowing fibers to start and terminate there. This will ensure that fibers like the cerebral peduncle are captured although not anatomically resolved. Cerebellar regions were not used given the same anatomical constraints and less well established atlases.

Third, the reproducibility measurement employed throughout the manuscript, i.e., the ICC, is attenuated if assumptions of normality are violated[37]. Thus, the ICC can underestimate reproducibility. This limitation may be instrumental in the context of the structural connectome, since link weights that do not conform to a normal distribution are those that are relatively unstable across many subjects (being therefore close to zero). These links are not homogeneously resolved across all subjects and the ICC identifies them as less reproducible. Nonetheless, it is important to notice that the reproducibility described here may in fact be mildly underestimated.

Fourth, we used the number of streamline to represent connectivity strength. However, DTI streamlines are mathematical constructs that may not necessarily represent connectivity strength [38]. In this context, our observations regarding the reproducibility of graph theory measures may be more relevant, since they disclose the reliability of the structural conformation rather than link wise weight.

Fifth, gray matter ROI segmentation was performed based on an anatomically defined atlas [17, 39]. Nonetheless, the division of the gray matter into ROIs is a semi-arbitrary process, which may not directly represent functional or histological boundaries. Thus, gray matter ROIs may be influenced by anatomical variability and may be inferior to connectivity based parcellation schemes that respect individual patterns of connectivity.

### Conclusions

In summary, in this study we described the reproducibility of structural connectome mapping as a function of DTI tractography approach, anatomical properties of structural links and MRI scanners. Our results demonstrate that connectome mapping is a largely reproducible technique, particularly as it related to the geometry of network architecture (measured by graph theory) but special attention should be devoted to methodological and anatomical aspects associated with lower reproducibility.

## Supporting Information

S1 Data All raw connectome matrices evaluated in this study are available in the compressed file “Connectome_data.zip”. Please refer to the README.txt file within the compressed folder for usage instructions.
(ZIP)

S1 Fig Native T1 images with overlaid anatomical ROIs, non-diffusion images, parcellation results, fiber tractography and connectome matrices for one representative subject across the 3 scanning sessions. The scale bars represent log(number of DTI streamlines).
(PNG)

S2 Fig Histograms demonstrating the distribution of ICCs. (TIFF)

S3 Fig Relationship between link-wise reproducibility across methods. Each element in the scatter plot indicates an anatomical link. (TIFF)

S4 Fig Relationship between each link’s ICC (x-axis) and its number of fibers tracked on average across all subjects (y-axis). (TIFF)

S5 Fig Relationship between the variability in the number of tracked fibers per subject and its reproducibility. (TIFF)

S1 Table Connectome links are ranked based on their reproducibility (ICC). The gray matter regions represent the connected nodes by each link, obtained from the Lausanne anatomical atlas, distributed as part of the Connectome Mapping Toolkit (http://www.connectome.ch). The white matter regions were extracted from the Johns Hopkins University DTI-based white matter atlas [40] and they represent the white matter area traversed by a centroid path corresponding to the center of mass of the fibers composing the link [41].
(DOCX)

## References

[1] Hagmann P, Cammoun L, Gigandet X, Meuli R, Honey CJ, Wedeen VJ, et al Mapping the structural core of human cerebral cortex. PLoS biology. 2008;6(7):e159 Epub 2008/07/04. 10.1371/journal.pbio.0060159 18597554 PMC2443193

[2] Mori S, van Zijl PC. Fiber tracking: principles and strategies—a technical review. NMR in biomedicine. 2002;15(7–8):468–80. Epub 2002/12/19. 10.1002/nbm.781 . 12489096

[3] Dauguet J, Peled S, Berezovskii V, Delzescaux T, Warfield SK, Born R, et al Comparison of fiber tracts derived from in-vivo DTI tractography with 3D histological neural tract tracer reconstruction on a macaque brain. Neuroimage. 2007;37(2):530–8. Epub 2007/07/03. 10.1016/j.neuroimage.2007.04.067 . 17604650

[4] Sporns O. The human connectome: origins and challenges. Neuroimage. 2013;80:53–61. Epub 2013/03/27. 10.1016/j.neuroimage.2013.03.023 . 23528922

[5] Hagmann P, Cammoun L, Gigandet X, Gerhard S, Grant PE, Wedeen V, et al MR connectomics: Principles and challenges. Journal of neuroscience methods. 2010;194(1):34–45. Epub 2010/01/26. 10.1016/j.jneumeth.2010.01.014 . 20096730

[6] Sporns O. From simple graphs to the connectome: networks in neuroimaging. Neuroimage. 2012;62(2):881–6. Epub 2011/10/04. 10.1016/j.neuroimage.2011.08.085 . 21964480

[7] Sporns O. The human connectome: a complex network. Ann N Y Acad Sci. 2011;1224:109–25. Epub 2011/01/22. 10.1111/j.1749-6632.2010.05888.x . 21251014

[8] Sporns O, Tononi G, Kotter R. The human connectome: A structural description of the human brain. PLoS computational biology. 2005;1(4):e42 Epub 2005/10/05. 10.1371/journal.pcbi.0010042 16201007 PMC1239902

[9] Leergaard TB, Hilgetag CC, Sporns O. Mapping the connectome: multi-level analysis of brain connectivity. Frontiers in neuroinformatics. 2012;6:14 Epub 2012/05/05. 10.3389/fninf.2012.00014 22557964 PMC3340894

[10] Zhang Z, Liao W, Chen H, Mantini D, Ding JR, Xu Q, et al Altered functional-structural coupling of large-scale brain networks in idiopathic generalized epilepsy. Brain. 2011;134(Pt 10):2912–28. 10.1093/brain/awr223 . 21975588

[11] Quan M, Lee SH, Kubicki M, Kikinis Z, Rathi Y, Seidman LJ, et al White matter tract abnormalities between rostral middle frontal gyrus, inferior frontal gyrus and striatum in first-episode schizophrenia. Schizophrenia research. 2013;145(1–3):1–10. Epub 2013/02/19. 10.1016/j.schres.2012.11.028 . 23415471 PMC4110910

[12] Lo CY, Wang PN, Chou KH, Wang J, He Y, Lin CP. Diffusion tensor tractography reveals abnormal topological organization in structural cortical networks in Alzheimer's disease. J Neurosci. 2010;30(50):16876–85. Epub 2010/12/17. 10.1523/JNEUROSCI.4136-10.2010 . 21159959 PMC6634928

[13] Richardson MP. Large scale brain models of epilepsy: dynamics meets connectomics. J Neurol Neurosurg Psychiatry. 2012;83(12):1238–48. Epub 2012/08/25. 10.1136/jnnp-2011-301944 . 22917671

[14] Wang JY, Abdi H, Bakhadirov K, Diaz-Arrastia R, Devous MD Sr. A comprehensive reliability assessment of quantitative diffusion tensor tractography. Neuroimage. 2012;60(2):1127–38. 10.1016/j.neuroimage.2011.12.062 22227883 PMC3468740

[15] Behrens TE, Woolrich MW, Jenkinson M, Johansen-Berg H, Nunes RG, Clare S, et al Characterization and propagation of uncertainty in diffusion-weighted MR imaging. Magnetic resonance in medicine: official journal of the Society of Magnetic Resonance in Medicine / Society of Magnetic Resonance in Medicine. 2003;50(5):1077–88. 10.1002/mrm.10609 . 14587019

[16] Dale AM, Fischl B, Sereno MI. Cortical surface-based analysis. I. Segmentation and surface reconstruction. Neuroimage. 1999;9(2):179–94. Epub 1999/02/05. 10.1006/nimg.1998.0395 . 9931268

[17] Fischl B, Dale AM. Measuring the thickness of the human cerebral cortex from magnetic resonance images. Proc Natl Acad Sci U S A. 2000;97(20):11050–5. Epub 2000/09/14. 10.1073/pnas.200033797 10984517 PMC27146

[18] Behrens TE, Berg HJ, Jbabdi S, Rushworth MF, Woolrich MW. Probabilistic diffusion tractography with multiple fibre orientations: What can we gain? Neuroimage. 2007;34(1):144–55. 10.1016/j.neuroimage.2006.09.018 . 17070705 PMC7116582

[19] Wang R, Benner T, Sorensen AG, Wedeen VJ. Diffusion Toolkit: A Software Package for Diffusion Imaging Data Processing and Tractography. Proc Intl Soc Mag Reson Med 2007.

[20] McGraw KOW, S.P. Forming inferences about some intraclass correlation coefficients. Psychological Methods. 1996;1(1):30–46.

[21] Magnotta VA, Matsui JT, Liu D, Johnson HJ, Long JD, Bolster BD Jr, et al Multicenter reliability of diffusion tensor imaging. Brain connectivity. 2012;2(6):345–55. Epub 2012/10/19. 10.1089/brain.2012.0112 23075313 PMC3623569

[22] Vollmar C, O'Muircheartaigh J, Barker GJ, Symms MR, Thompson P, Kumari V, et al Identical, but not the same: intra-site and inter-site reproducibility of fractional anisotropy measures on two 3.0T scanners. Neuroimage. 2010;51(4):1384–94. Epub 2010/03/27. 10.1016/j.neuroimage.2010.03.046 20338248 PMC3163823

[23] Heiervang E, Behrens TE, Mackay CE, Robson MD, Johansen-Berg H. Between session reproducibility and between subject variability of diffusion MR and tractography measures. Neuroimage. 2006;33(3):867–77. Epub 2006/09/27. 10.1016/j.neuroimage.2006.07.037 . 17000119

[24] Besseling RM, Jansen JF, Overvliet GM, Vaessen MJ, Braakman HM, Hofman PA, et al Tract specific reproducibility of tractography based morphology and diffusion metrics. PloS one. 2012;7(4):e34125 10.1371/journal.pone.0034125 22485157 PMC3317780

[25] Danielian LE, Iwata NK, Thomasson DM, Floeter MK. Reliability of fiber tracking measurements in diffusion tensor imaging for longitudinal study. NeuroImage. 2010;49(2):1572–80. 10.1016/j.neuroimage.2009.08.062 19744567 PMC2789889

[26] Buchanan CR, Pernet CR, Gorgolewski KJ, Storkey AJ, Bastin ME. Test-retest reliability of structural brain networks from diffusion MRI. NeuroImage. 2014;86:231–43. 10.1016/j.neuroimage.2013.09.054 . 24096127

[27] Duda JT, Cook PA, Gee JC. Reproducibility of graph metrics of human brain structural networks. Frontiers in neuroinformatics. 2014;8:46 10.3389/fninf.2014.00046 24847245 PMC4019854

[28] Cheng H, Wang Y, Sheng J, Kronenberger WG, Mathews VP, Hummer TA, et al Characteristics and variability of structural networks derived from diffusion tensor imaging. NeuroImage. 2012;61(4):1153–64. 10.1016/j.neuroimage.2012.03.036 22450298 PMC3500617

[29] Vaessen MJ, Hofman PA, Tijssen HN, Aldenkamp AP, Jansen JF, Backes WH. The effect and reproducibility of different clinical DTI gradient sets on small world brain connectivity measures. Neuroimage. 2010;51(3):1106–16. 10.1016/j.neuroimage.2010.03.011 . 20226864

[30] Kaiser M. The potential of the human connectome as a biomarker of brain disease. Frontiers in human neuroscience. 2013;7:484 10.3389/fnhum.2013.00484 23966935 PMC3744009

[31] Fornito A, Zalesky A, Breakspear M. The connectomics of brain disorders. Nature reviews Neuroscience. 2015;16(3):159–72. 10.1038/nrn3901 . 25697159

[32] Deco G, Kringelbach ML. Great expectations: using whole-brain computational connectomics for understanding neuropsychiatric disorders. Neuron. 2014;84(5):892–905. 10.1016/j.neuron.2014.08.034 . 25475184

[33] Griffa A, Baumann PS, Thiran JP, Hagmann P. Structural connectomics in brain diseases. Neuroimage. 2013;80:515–26. 10.1016/j.neuroimage.2013.04.056 . 23623973

[34] Tabesh A, Jensen JH, Ardekani BA, Helpern JA. Estimation of tensors and tensor-derived measures in diffusional kurtosis imaging. Magnetic resonance in medicine: official journal of the Society of Magnetic Resonance in Medicine / Society of Magnetic Resonance in Medicine. 2011;65(3):823–36. Epub 2011/02/22. 10.1002/mrm.22655 21337412 PMC3042509

[35] Lee CY, Tabesh A, Nesland T, Jensen JH, Helpern JA, Spampinato MV, et al Human brain asymmetry in microstructural connectivity demonstrated by diffusional kurtosis imaging. Brain research. 2014;1588:73–80. 10.1016/j.brainres.2014.09.002 . 25239477 PMC4495905

[36] Wedeen VJ, Wang RP, Schmahmann JD, Benner T, Tseng WY, Dai G, et al Diffusion spectrum magnetic resonance imaging (DSI) tractography of crossing fibers. NeuroImage. 2008;41(4):1267–77. Epub 2008/05/23. 10.1016/j.neuroimage.2008.03.036 . 18495497

[37] Osborne JW. Best practices in quantitative methods Thousand Oaks, Calif.: Sage Publications; 2008 xii, 596 p. p.

[38] Fornito A, Zalesky A, Breakspear M. Graph analysis of the human connectome: promise, progress, and pitfalls. NeuroImage. 2013;80:426–44. 10.1016/j.neuroimage.2013.04.087 . 23643999

[39] Gerhard S, Daducci A, Lemkaddem A, Meuli R, Thiran JP, Hagmann P. The connectome viewer toolkit: an open source framework to manage, analyze, and visualize connectomes. Frontiers in neuroinformatics. 2011;5:3 10.3389/fninf.2011.00003 21713110 PMC3112315

[40] Hua K, Zhang J, Wakana S, Jiang H, Li X, Reich DS, et al Tract probability maps in stereotaxic spaces: analyses of white matter anatomy and tract-specific quantification. Neuroimage. 2008;39(1):336–47. 10.1016/j.neuroimage.2007.07.053 17931890 PMC2724595

[41] Garyfallidis E, Brett M, Correia MM, Williams GB, Nimmo-Smith I. QuickBundles, a Method for Tractography Simplification. Frontiers in neuroscience. 2012;6:175 Epub 2012/12/19. 10.3389/fnins.2012.00175 23248578 PMC3518823

