# Supplementary figures and images for: Reproducibility of the Structural Brain Connectome Derived from Diffusion Tensor Imaging
Source: PLoS One. 2015 Sep 2;10(9):e0135247. doi: 10.1371/journal.pone.0135247 (PMC4557836; doi:10.1371/journal.pone.0135247)

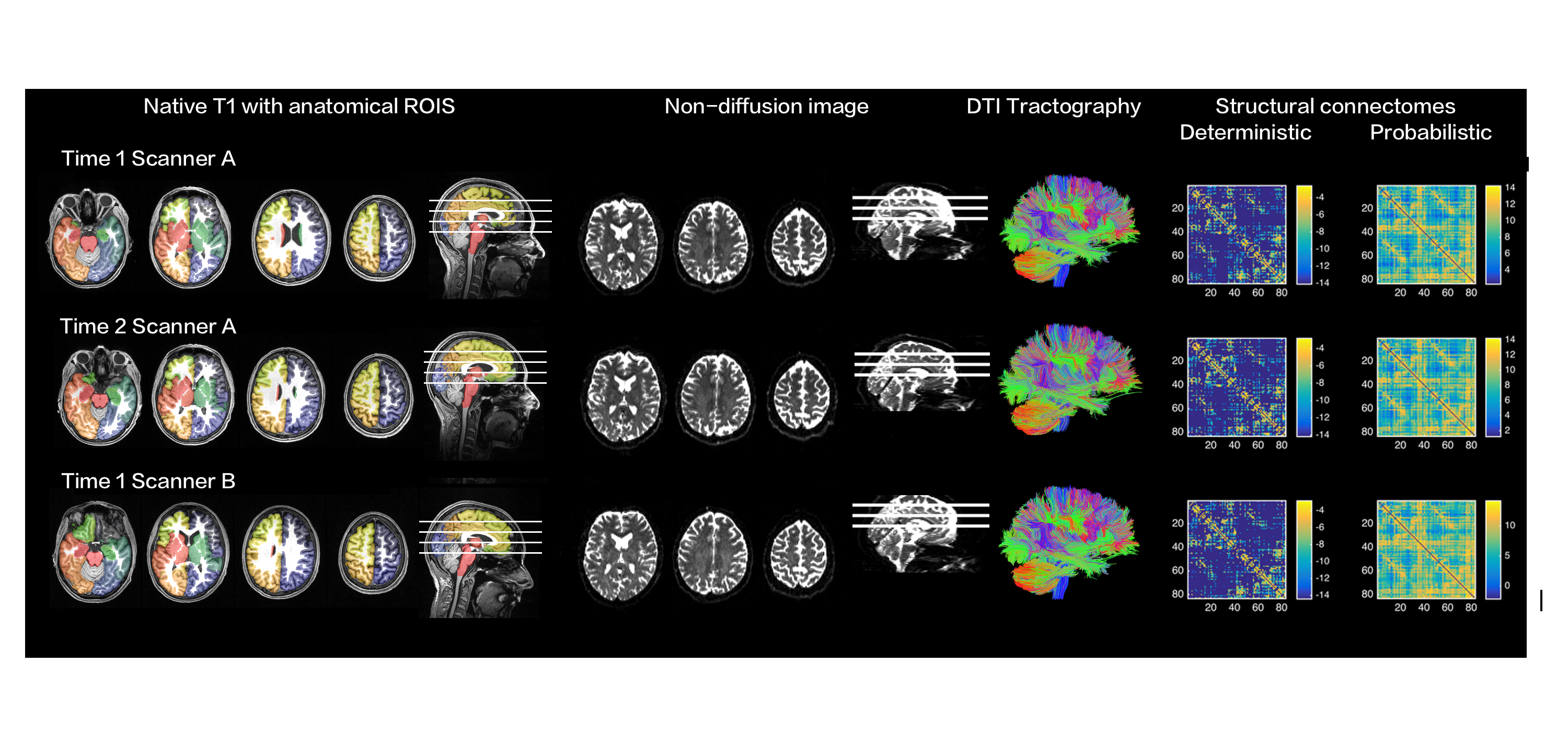

Supplement: S1 Fig — The scale bars represent log(number of DTI streamlines). (PNG) [file pone.0135247.s002.png]

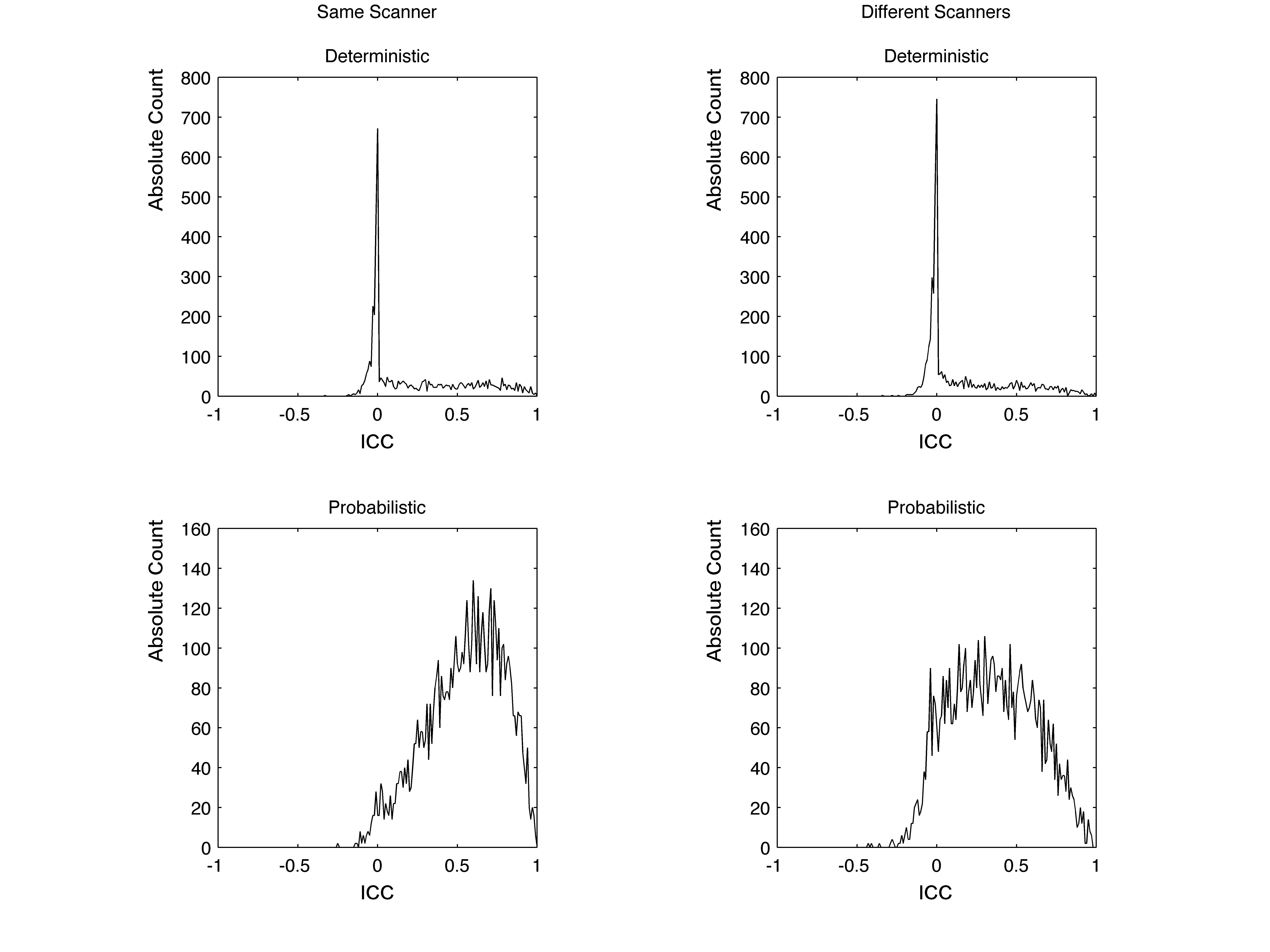

Supplement: S2 Fig — (TIFF) [file pone.0135247.s003.tiff]

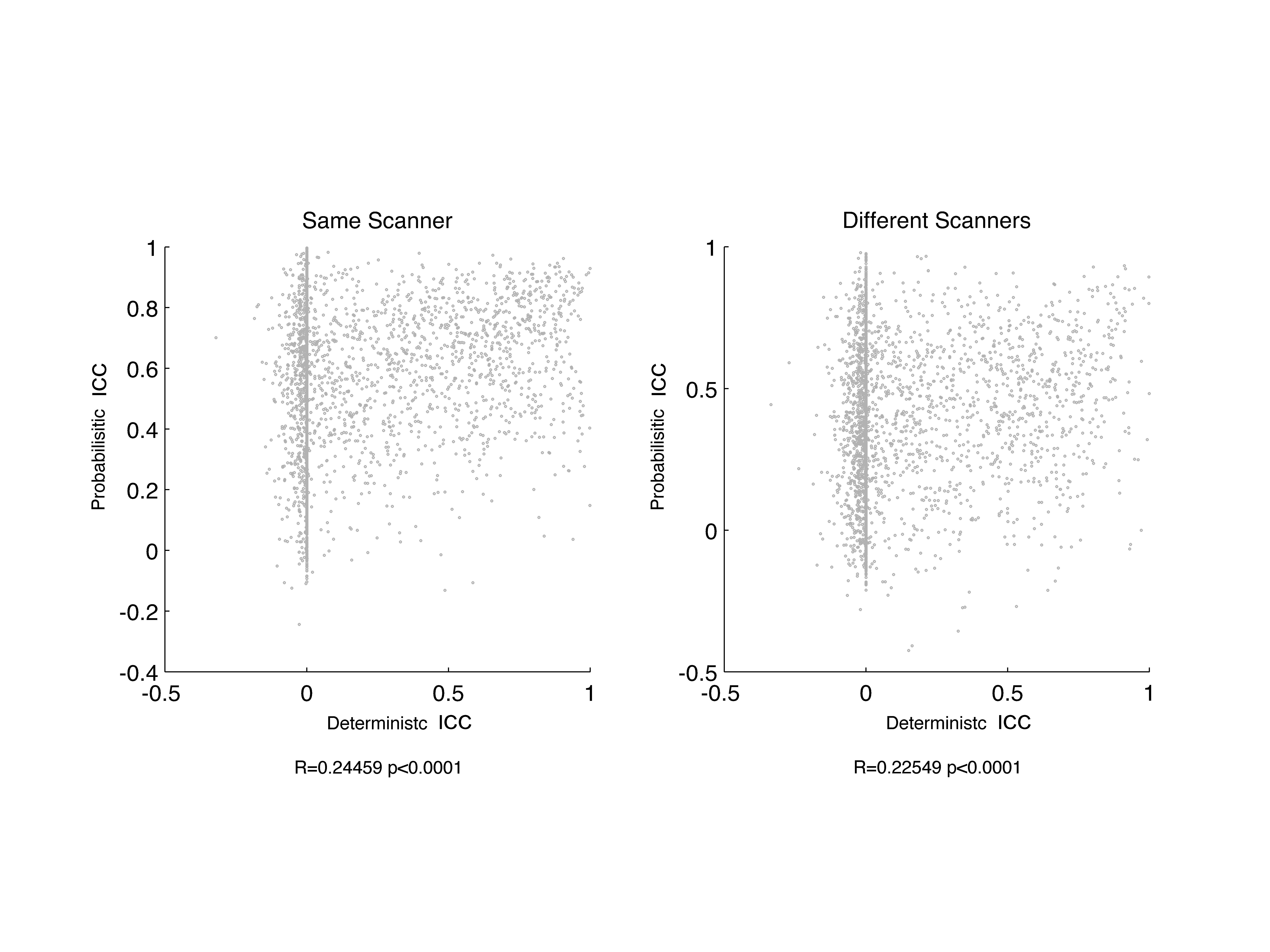

Supplement: S3 Fig — (TIFF) [file pone.0135247.s004.tiff]

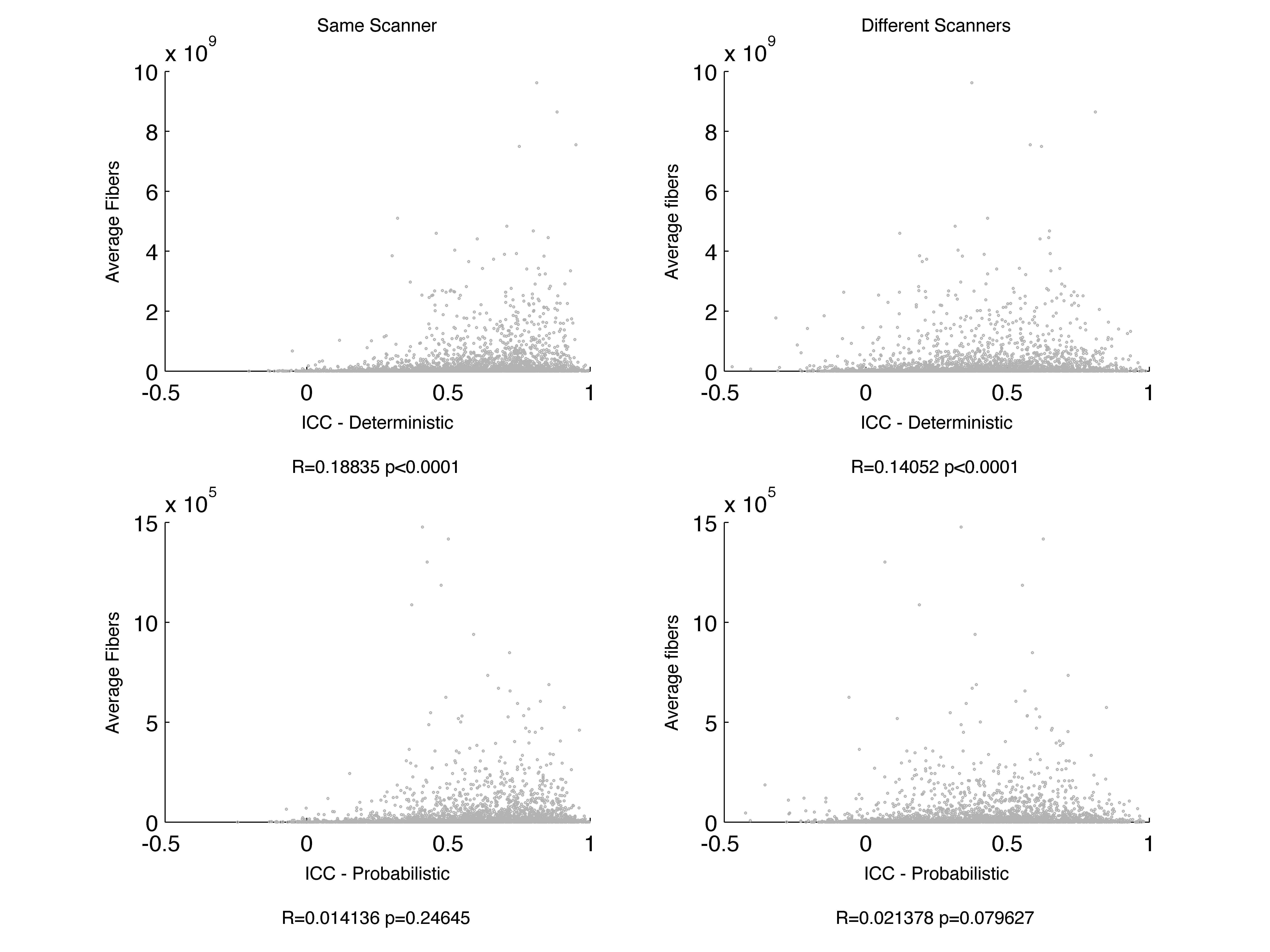

Supplement: S4 Fig — (TIFF) [file pone.0135247.s005.tiff]

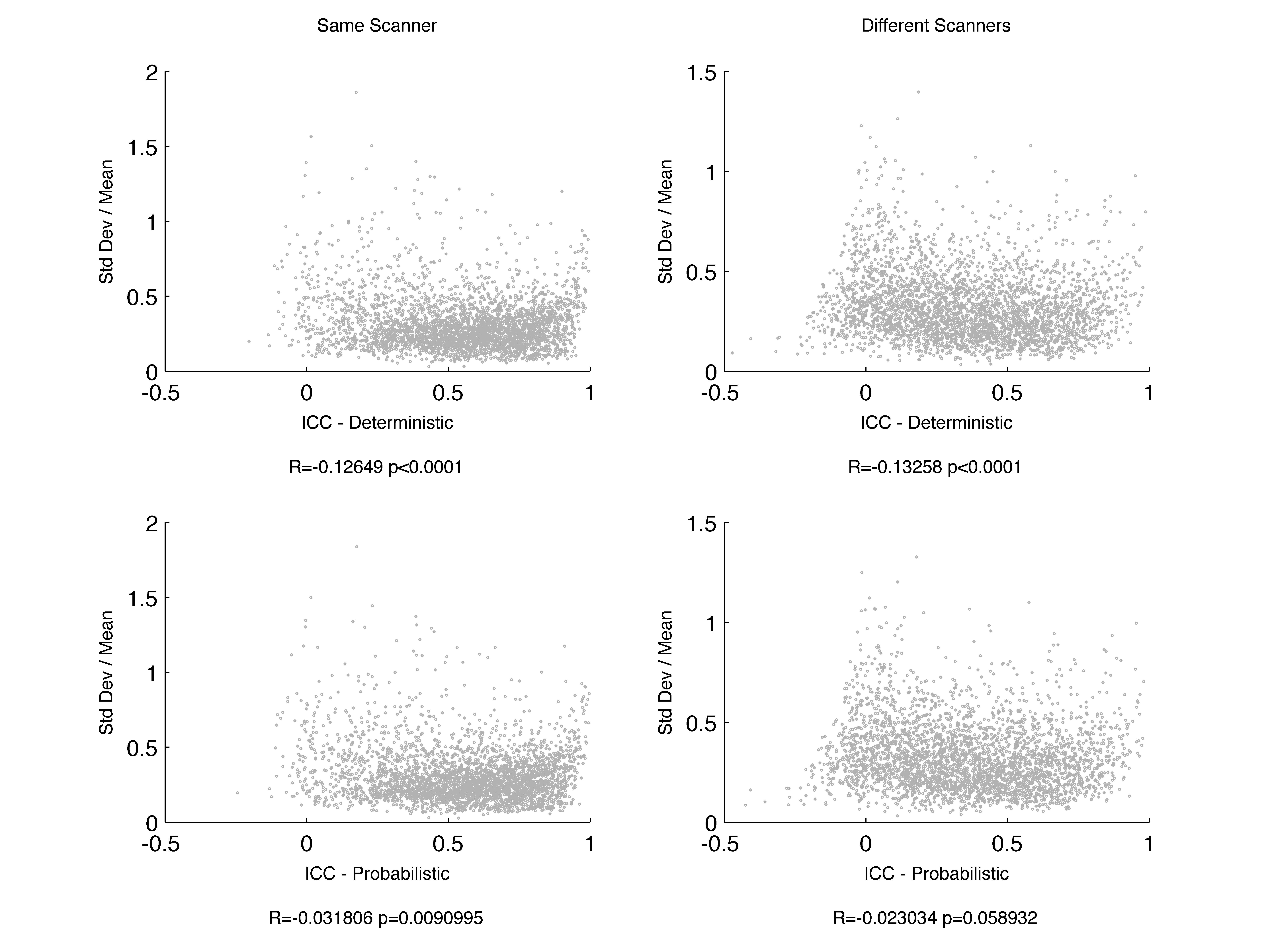

Supplement: S5 Fig — (TIFF) [file pone.0135247.s006.tiff]
