# Supplementary material for: Reproducibility of the Structural Brain Connectome Derived from Diffusion Tensor Imaging
Source: PLoS One. 2015 Sep 2;10(9):e0135247. doi: 10.1371/journal.pone.0135247 (PMC4557836; doi:10.1371/journal.pone.0135247)
Supplement: S1 Table — The gray matter regions represent the connected nodes by each link, obtained from the Lausanne anatomical atlas, distributed as part of the Connectome Mapping Toolkit (http://www.connectome.ch). The white matter regions were extracted from the Johns Hopkins University DTI-based white matter atlas [40] and they represent the white matter area traversed by a centroid path corresponding to the center of mass of the fibers composing the link [41]. (DOCX) [file pone.0135247.s007.docx]

***Supplementary Table 1-*** Connectome links are ranked based on their reproducibility (ICC). The gray matter regions represent the connected nodes by each link, obtained from the Lausanne anatomical atlas, distributed as part of the Connectome Mapping Toolkit (<http://www.connectome.ch>). The white matter regions were extracted from the Johns Hopkins University DTI-based white matter atlas (Hua et al., 2008) and they represent the white matter area traversed by a centroid path corresponding to the center of mass of the fibers composing the link (Garyfallidis et al., 2012).

| ***Deterministic Tractograhy (Same Scanner)*** | | | |
| --- | --- | --- | --- |
| Gray matter region | Gray matter region | ICC | White matter tract |
| Rightsuperiorfrontal | RightPutamen | 0.986408 | Anterior corona radiata |
| RightPutamen | Rightsuperiorfrontal | 0.986408 | Anterior corona radiata |
| Rightparsopercularis | Rightsuperiorparietal | 0.973823 | Superior Longitudinal Fasciculus |
| Rightsuperiorparietal | Rightparsopercularis | 0.973823 | Superior Longitudinal Fasciculus |
| Rightprecentral | Rightbankssts | 0.969566 | Fornix (cres) / Stria terminalis |
| Rightbankssts | Rightprecentral | 0.969566 | Fornix (cres) / Stria terminalis |
| Rightpostcentral | Rightbankssts | 0.963182 | Fornix (cres) / Stria terminalis |
| Rightbankssts | Rightpostcentral | 0.963182 | Fornix (cres) / Stria terminalis |
| Rightcaudalmiddlefrontal | Rightmiddletemporal | 0.960102 | Fornix (cres) / Stria terminalis |
| Rightmiddletemporal | Rightcaudalmiddlefrontal | 0.960102 | Fornix (cres) / Stria terminalis |
| Leftlateralorbitofrontal | LeftAmygdala | 0.956483 | External capsule |
| LeftAmygdala | Leftlateralorbitofrontal | 0.956483 | External capsule |
| RightPutamen | Leftinferiortemporal | 0.943792 | Body of corpus callosum |
| Leftinferiortemporal | RightPutamen | 0.943792 | Body of corpus callosum |
| LeftCaudate | BrainStem | 0.921175 | Anterior limb of internal capsule |
| BrainStem | LeftCaudate | 0.921175 | Anterior limb of internal capsule |
| Rightsuperiorfrontal | RightThalamusProper | 0.91249 | Anterior corona radiata |
| RightThalamusProper | Rightsuperiorfrontal | 0.91249 | Anterior corona radiata |
| Rightprecentral | LeftThalamusProper | 0.909794 | Genu of corpus callosum |
| LeftThalamusProper | Rightprecentral | 0.909794 | Genu of corpus callosum |
| Leftrostralmiddlefrontal | LeftPutamen | 0.908534 | Anterior corona radiata |
| LeftPutamen | Leftrostralmiddlefrontal | 0.908534 | Anterior corona radiata |
| Rightprecentral | Rightmiddletemporal | 0.906237 | Fornix (cres) / Stria terminalis |
| Rightmiddletemporal | Rightprecentral | 0.906237 | Fornix (cres) / Stria terminalis |
| Rightparacentral | BrainStem | 0.904208 | Anterior limb of internal capsule |
| BrainStem | Rightparacentral | 0.904208 | Anterior limb of internal capsule |
| Leftparsorbitalis | LeftPallidum | 0.897822 | Anterior limb of internal capsule |
| LeftPallidum | Leftparsorbitalis | 0.897822 | Anterior limb of internal capsule |
| Leftfrontalpole | LeftPutamen | 0.896247 | Anterior corona radiata |
| LeftPutamen | Leftfrontalpole | 0.896247 | Anterior corona radiata |
| Rightcaudalmiddlefrontal | Rightsupramarginal | 0.878838 | Fornix (cres) / Stria terminalis |
| Rightsupramarginal | Rightcaudalmiddlefrontal | 0.878838 | Fornix (cres) / Stria terminalis |
| Rightprecentral | Leftprecentral | 0.877964 | Genu of corpus callosum |
| Leftprecentral | Rightprecentral | 0.877964 | Genu of corpus callosum |
| Leftrostralmiddlefrontal | BrainStem | 0.866795 | Anterior limb of internal capsule |
| BrainStem | Leftrostralmiddlefrontal | 0.866795 | Anterior limb of internal capsule |
| Rightsuperiorfrontal | Leftsuperiorfrontal | 0.866096 | Genu of corpus callosum |
| Leftsuperiorfrontal | Rightsuperiorfrontal | 0.866096 | Genu of corpus callosum |
| RightHippocampus | Leftsuperiorparietal | 0.861597 | Body of corpus callosum |
| Leftsuperiorparietal | RightHippocampus | 0.861597 | Body of corpus callosum |
| Rightparsorbitalis | RightThalamusProper | 0.861583 | Cerebral peduncle |
| RightThalamusProper | Rightparsorbitalis | 0.861583 | Cerebral peduncle |
| Rightprecuneus | Leftsuperiorparietal | 0.857902 | Body of corpus callosum |
| Leftsuperiorparietal | Rightprecuneus | 0.857902 | Body of corpus callosum |
| Rightpostcentral | Rightmiddletemporal | 0.856438 | Fornix (cres) / Stria terminalis |
| Rightmiddletemporal | Rightpostcentral | 0.856438 | Fornix (cres) / Stria terminalis |
| Rightprecuneus | BrainStem | 0.854773 | Anterior limb of internal capsule |
| BrainStem | Rightprecuneus | 0.854773 | Anterior limb of internal capsule |
| Leftparstriangularis | LeftCaudate | 0.853453 | Anterior corona radiata |
| LeftCaudate | Leftparstriangularis | 0.853453 | Anterior corona radiata |
| Rightposteriorcingulate | Leftisthmuscingulate | 0.837022 | Genu of corpus callosum |
| Leftisthmuscingulate | Rightposteriorcingulate | 0.837022 | Genu of corpus callosum |
| Rightprecuneus | Leftprecentral | 0.831334 | Corpus callosum |
| Leftprecentral | Rightprecuneus | 0.831334 | Corpus callosum |
| Rightsuperiorparietal | LeftHippocampus | 0.823736 | Body of corpus callosum |
| LeftHippocampus | Rightsuperiorparietal | 0.823736 | Body of corpus callosum |
| RightPutamen | BrainStem | 0.823152 | Anterior limb of internal capsule |
| BrainStem | RightPutamen | 0.823152 | Anterior limb of internal capsule |
| Rightparsopercularis | Rightsupramarginal | 0.810344 | Fornix (cres) / Stria terminalis |
| Rightsupramarginal | Rightparsopercularis | 0.810344 | Fornix (cres) / Stria terminalis |
| Leftcaudalmiddlefrontal | LeftPutamen | 0.809384 | External capsule |
| LeftPutamen | Leftcaudalmiddlefrontal | 0.809384 | External capsule |
| Rightparacentral | Leftparacentral | 0.806756 | Genu of corpus callosum |
| Leftparacentral | Rightparacentral | 0.806756 | Genu of corpus callosum |
| Rightcaudalanteriorcingulate | RightThalamusProper | 0.804841 | Cerebral peduncle |
| RightThalamusProper | Rightcaudalanteriorcingulate | 0.804841 | Cerebral peduncle |
| Rightparstriangularis | Rightprecentral | 0.801691 | Fornix (cres) / Stria terminalis |
| Rightprecentral | Rightparstriangularis | 0.801691 | Fornix (cres) / Stria terminalis |
| Leftparsopercularis | LeftPutamen | 0.797426 | Anterior corona radiata |
| LeftPutamen | Leftparsopercularis | 0.797426 | Anterior corona radiata |
| Leftisthmuscingulate | Leftprecuneus | 0.793764 | Body of corpus callosum |
| Leftprecuneus | Leftisthmuscingulate | 0.793764 | Body of corpus callosum |
| Rightmiddletemporal | RightPutamen | 0.793743 | Fornix (cres) / Stria terminalis |
| RightPutamen | Rightmiddletemporal | 0.793743 | Fornix (cres) / Stria terminalis |
| Rightcaudalanteriorcingulate | LeftPallidum | 0.793045 | Anterior corona radiata |
| LeftPallidum | Rightcaudalanteriorcingulate | 0.793045 | Anterior corona radiata |
| Rightcaudalmiddlefrontal | BrainStem | 0.789515 | Anterior limb of internal capsule |
| BrainStem | Rightcaudalmiddlefrontal | 0.789515 | Anterior limb of internal capsule |
| Leftrostralmiddlefrontal | LeftCaudate | 0.789152 | Anterior corona radiata |
| LeftCaudate | Leftrostralmiddlefrontal | 0.789152 | Anterior corona radiata |
| Rightcaudalmiddlefrontal | RightThalamusProper | 0.788491 | Anterior corona radiata |
| RightThalamusProper | Rightcaudalmiddlefrontal | 0.788491 | Anterior corona radiata |
| Rightcaudalmiddlefrontal | Leftsuperiorfrontal | 0.787725 | Genu of corpus callosum |
| Leftsuperiorfrontal | Rightcaudalmiddlefrontal | 0.787725 | Genu of corpus callosum |
| RightHippocampus | RightAmygdala | 0.786797 | Cingulum (hippocampus) |
| RightAmygdala | RightHippocampus | 0.786797 | Cingulum (hippocampus) |
| Rightsuperiorfrontal | BrainStem | 0.78457 | Anterior limb of internal capsule |
| BrainStem | Rightsuperiorfrontal | 0.78457 | Anterior limb of internal capsule |
| Rightprecentral | Rightsupramarginal | 0.783404 | Fornix (cres) / Stria terminalis |
| Rightsupramarginal | Rightprecentral | 0.783404 | Fornix (cres) / Stria terminalis |
| Leftparstriangularis | LeftPutamen | 0.783354 | Anterior corona radiata |
| LeftPutamen | Leftparstriangularis | 0.783354 | Anterior corona radiata |
| Rightinferiorparietal | Rightinferiortemporal | 0.782176 | Fornix (cres) / Stria terminalis |
| Rightinferiortemporal | Rightinferiorparietal | 0.782176 | Fornix (cres) / Stria terminalis |
| Rightprecentral | RightPutamen | 0.779142 | Anterior corona radiata |
| RightPutamen | Rightprecentral | 0.779142 | Anterior corona radiata |
| Rightsuperiorfrontal | LeftCaudate | 0.766587 | Genu of corpus callosum |
| LeftCaudate | Rightsuperiorfrontal | 0.766587 | Genu of corpus callosum |
| Rightparsopercularis | Rightinferiorparietal | 0.763211 | Fornix (cres) / Stria terminalis |
| Rightinferiorparietal | Rightparsopercularis | 0.763211 | Fornix (cres) / Stria terminalis |
| Rightsupramarginal | Rightmiddletemporal | 0.762566 | Fornix (cres) / Stria terminalis |
| Rightmiddletemporal | Rightsupramarginal | 0.762566 | Fornix (cres) / Stria terminalis |
| Leftposteriorcingulate | Leftprecuneus | 0.758486 | Cingulum (cingulate gyrus) |
| Leftprecuneus | Leftposteriorcingulate | 0.758486 | Cingulum (cingulate gyrus) |
| Rightinferiorparietal | RightPutamen | 0.757846 | Fornix (cres) / Stria terminalis |
| RightPutamen | Rightinferiorparietal | 0.757846 | Fornix (cres) / Stria terminalis |
| Leftcaudalanteriorcingulate | LeftAccumbensarea | 0.756959 | Anterior corona radiata |
| LeftAccumbensarea | Leftcaudalanteriorcingulate | 0.756959 | Anterior corona radiata |
| Leftparstriangularis | Leftsuperiortemporal | 0.752879 | External capsule |
| Leftsuperiortemporal | Leftparstriangularis | 0.752879 | External capsule |
| Rightsuperiorparietal | Leftsuperiorparietal | 0.751355 | Body of corpus callosum |
| Leftsuperiorparietal | Rightsuperiorparietal | 0.751355 | Body of corpus callosum |
| Rightpostcentral | BrainStem | 0.749325 | Anterior limb of internal capsule |
| BrainStem | Rightpostcentral | 0.749325 | Anterior limb of internal capsule |
| Rightisthmuscingulate | Leftprecuneus | 0.748089 | Body of corpus callosum |
| Leftprecuneus | Rightisthmuscingulate | 0.748089 | Body of corpus callosum |
| Rightcaudalanteriorcingulate | Leftcaudalanteriorcingulate | 0.747661 | Cingulum (cingulate gyrus) |
| Leftcaudalanteriorcingulate | Rightcaudalanteriorcingulate | 0.747661 | Cingulum (cingulate gyrus) |
| Rightsuperiorfrontal | Rightcaudalanteriorcingulate | 0.747514 | External capsule |
| Rightcaudalanteriorcingulate | Rightsuperiorfrontal | 0.747514 | External capsule |
| Rightsuperiorparietal | BrainStem | 0.74124 | Anterior limb of internal capsule |
| BrainStem | Rightsuperiorparietal | 0.74124 | Anterior limb of internal capsule |
| Rightsuperiorfrontal | Leftprecentral | 0.739333 | Genu of corpus callosum |
| Leftprecentral | Rightsuperiorfrontal | 0.739333 | Genu of corpus callosum |
| Rightpostcentral | RightPutamen | 0.734612 | Anterior corona radiata |
| RightPutamen | Rightpostcentral | 0.734612 | Anterior corona radiata |
| Rightsuperiorfrontal | RightPallidum | 0.732932 | Anterior corona radiata |
| RightPallidum | Rightsuperiorfrontal | 0.732932 | Anterior corona radiata |
| Rightparacentral | RightCaudate | 0.728971 | Anterior corona radiata |
| RightCaudate | Rightparacentral | 0.728971 | Anterior corona radiata |
| Rightparacentral | RightThalamusProper | 0.723631 | Anterior corona radiata |
| RightThalamusProper | Rightparacentral | 0.723631 | Anterior corona radiata |
| Rightsuperiorfrontal | Leftinsula | 0.723612 | Genu of corpus callosum |
| Leftinsula | Rightsuperiorfrontal | 0.723612 | Genu of corpus callosum |
| Rightparacentral | Leftsuperiorfrontal | 0.720265 | Genu of corpus callosum |
| Leftsuperiorfrontal | Rightparacentral | 0.720265 | Genu of corpus callosum |
| Leftmedialorbitofrontal | LeftPutamen | 0.717165 | Anterior corona radiata |
| LeftPutamen | Leftmedialorbitofrontal | 0.717165 | Anterior corona radiata |
| Leftparstriangularis | Leftinsula | 0.715472 | Anterior corona radiata |
| Leftinsula | Leftparstriangularis | 0.715472 | Anterior corona radiata |
| RightPallidum | Leftinsula | 0.703467 | Genu of corpus callosum |
| Leftinsula | RightPallidum | 0.703467 | Genu of corpus callosum |
| LeftThalamusProper | LeftCaudate | 0.701416 | Anterior limb of internal capsule |
| LeftCaudate | LeftThalamusProper | 0.701416 | Anterior limb of internal capsule |
| Rightparacentral | Rightposteriorcingulate | 0.700758 | Anterior corona radiata |
| Rightposteriorcingulate | Rightparacentral | 0.700758 | Anterior corona radiata |
| Leftsuperiorfrontal | Leftcaudalanteriorcingulate | 0.700145 | Cingulum (cingulate gyrus) |
| Leftcaudalanteriorcingulate | Leftsuperiorfrontal | 0.700145 | Cingulum (cingulate gyrus) |
| Rightprecuneus | RightHippocampus | 0.696974 | Body of corpus callosum |
| RightHippocampus | Rightprecuneus | 0.696974 | Body of corpus callosum |
| RightThalamusProper | RightPutamen | 0.694408 | Anterior limb of internal capsule |
| RightPutamen | RightThalamusProper | 0.694408 | Anterior limb of internal capsule |
| Leftisthmuscingulate | Leftcuneus | 0.693129 | Body of corpus callosum |
| Leftcuneus | Leftisthmuscingulate | 0.693129 | Body of corpus callosum |
| Rightparacentral | Leftposteriorcingulate | 0.692783 | Genu of corpus callosum |
| Leftposteriorcingulate | Rightparacentral | 0.692783 | Genu of corpus callosum |
| Rightisthmuscingulate | RightHippocampus | 0.692604 | Cingulum (cingulate gyrus) |
| RightHippocampus | Rightisthmuscingulate | 0.692604 | Cingulum (cingulate gyrus) |
| Rightparstriangularis | RightThalamusProper | 0.689061 | Cerebral peduncle |
| RightThalamusProper | Rightparstriangularis | 0.689061 | Cerebral peduncle |
| Rightisthmuscingulate | Rightprecuneus | 0.687962 | Body of corpus callosum |
| Rightprecuneus | Rightisthmuscingulate | 0.687962 | Body of corpus callosum |
| Rightposteriorcingulate | Leftparacentral | 0.686985 | Genu of corpus callosum |
| Leftparacentral | Rightposteriorcingulate | 0.686985 | Genu of corpus callosum |
| RightCaudate | RightPutamen | 0.685222 | Cerebral peduncle |
| RightPutamen | RightCaudate | 0.685222 | Cerebral peduncle |
| Leftinferiortemporal | LeftPutamen | 0.682819 | External capsule |
| LeftPutamen | Leftinferiortemporal | 0.682819 | External capsule |
| Rightsuperiorparietal | Leftprecuneus | 0.682032 | Body of corpus callosum |
| Leftprecuneus | Rightsuperiorparietal | 0.682032 | Body of corpus callosum |
| Leftparsorbitalis | LeftCaudate | 0.680904 | Anterior corona radiata |
| LeftCaudate | Leftparsorbitalis | 0.680904 | Anterior corona radiata |
| Leftinferiortemporal | Leftinsula | 0.680252 | External capsule |
| Leftinsula | Leftinferiortemporal | 0.680252 | External capsule |
| Leftsuperiorparietal | LeftHippocampus | 0.679979 | Body of corpus callosum |
| LeftHippocampus | Leftsuperiorparietal | 0.679979 | Body of corpus callosum |
| Rightinferiortemporal | RightThalamusProper | 0.675283 | Cingulum (hippocampus) |
| RightThalamusProper | Rightinferiortemporal | 0.675283 | Cingulum (hippocampus) |
| Rightprecentral | BrainStem | 0.675117 | Anterior limb of internal capsule |
| BrainStem | Rightprecentral | 0.675117 | Anterior limb of internal capsule |
| RightThalamusProper | RightPallidum | 0.673697 | Anterior limb of internal capsule |
| RightPallidum | RightThalamusProper | 0.673697 | Anterior limb of internal capsule |
| Rightposteriorcingulate | Leftprecuneus | 0.672538 | Cingulum (cingulate gyrus) |
| Leftprecuneus | Rightposteriorcingulate | 0.672538 | Cingulum (cingulate gyrus) |
| Rightparsopercularis | Rightbankssts | 0.665706 | Fornix (cres) / Stria terminalis |
| Rightbankssts | Rightparsopercularis | 0.665706 | Fornix (cres) / Stria terminalis |
| Rightmiddletemporal | BrainStem | 0.664065 | Anterior limb of internal capsule |
| BrainStem | Rightmiddletemporal | 0.664065 | Anterior limb of internal capsule |
| Rightsuperiorfrontal | Rightprecuneus | 0.663375 | External capsule |
| Rightprecuneus | Rightsuperiorfrontal | 0.663375 | External capsule |
| Rightpostcentral | RightHippocampus | 0.661002 | Anterior limb of internal capsule |
| RightHippocampus | Rightpostcentral | 0.661002 | Anterior limb of internal capsule |
| Rightprecentral | RightCaudate | 0.660674 | Anterior corona radiata |
| RightCaudate | Rightprecentral | 0.660674 | Anterior corona radiata |
| Rightisthmuscingulate | Rightlingual | 0.658495 | Cingulum (cingulate gyrus) |
| Rightlingual | Rightisthmuscingulate | 0.658495 | Cingulum (cingulate gyrus) |
| Rightparsopercularis | Rightmiddletemporal | 0.655794 | Fornix (cres) / Stria terminalis |
| Rightmiddletemporal | Rightparsopercularis | 0.655794 | Fornix (cres) / Stria terminalis |
| Rightprecentral | Leftparacentral | 0.652741 | Genu of corpus callosum |
| Leftparacentral | Rightprecentral | 0.652741 | Genu of corpus callosum |
| ***Deterministic Tractograhy (Different Scanners)*** | | | |
| Leftlateralorbitofrontal | LeftAmygdala | 0.928524 | External capsule |
| LeftAmygdala | Leftlateralorbitofrontal | 0.928524 | External capsule |
| Rightsuperiorfrontal | RightPutamen | 0.881378 | Anterior corona radiata |
| RightPutamen | Rightsuperiorfrontal | 0.881378 | Anterior corona radiata |
| Rightprecentral | Rightmiddletemporal | 0.8812 | Fornix (cres) / Stria terminalis |
| Rightmiddletemporal | Rightprecentral | 0.8812 | Fornix (cres) / Stria terminalis |
| Rightcaudalmiddlefrontal | Rightinferiorparietal | 0.874575 | Fornix (cres) / Stria terminalis |
| Rightinferiorparietal | Rightcaudalmiddlefrontal | 0.874575 | Fornix (cres) / Stria terminalis |
| Rightparacentral | RightCaudate | 0.870214 | Anterior corona radiata |
| RightCaudate | Rightparacentral | 0.870214 | Anterior corona radiata |
| Rightparsopercularis | Rightmiddletemporal | 0.863467 | Fornix (cres) / Stria terminalis |
| Rightmiddletemporal | Rightparsopercularis | 0.863467 | Fornix (cres) / Stria terminalis |
| Rightpostcentral | RightPutamen | 0.859098 | Anterior corona radiata |
| RightPutamen | Rightpostcentral | 0.859098 | Anterior corona radiata |
| Rightprecuneus | RightHippocampus | 0.856907 | Body of corpus callosum |
| RightHippocampus | Rightprecuneus | 0.856907 | Body of corpus callosum |
| Rightprecentral | Rightbankssts | 0.855811 | Fornix (cres) / Stria terminalis |
| Rightbankssts | Rightprecentral | 0.855811 | Fornix (cres) / Stria terminalis |
| Rightisthmuscingulate | Leftprecuneus | 0.840356 | Body of corpus callosum |
| Leftprecuneus | Rightisthmuscingulate | 0.840356 | Body of corpus callosum |
| Rightcaudalmiddlefrontal | BrainStem | 0.831454 | Anterior limb of internal capsule |
| BrainStem | Rightcaudalmiddlefrontal | 0.831454 | Anterior limb of internal capsule |
| Rightsuperiorfrontal | Leftcaudalmiddlefrontal | 0.819831 | Cingulum (cingulate gyrus) |
| Leftcaudalmiddlefrontal | Rightsuperiorfrontal | 0.819831 | Cingulum (cingulate gyrus) |
| Leftparsopercularis | LeftPutamen | 0.817791 | Anterior corona radiata |
| LeftPutamen | Leftparsopercularis | 0.817791 | Anterior corona radiata |
| RightPallidum | LeftPallidum | 0.812648 | Anterior corona radiata |
| LeftPallidum | RightPallidum | 0.812648 | Anterior corona radiata |
| Rightmiddletemporal | RightPutamen | 0.810389 | Fornix (cres) / Stria terminalis |
| RightPutamen | Rightmiddletemporal | 0.810389 | Fornix (cres) / Stria terminalis |
| Leftparsopercularis | LeftThalamusProper | 0.798998 | Anterior limb of internal capsule |
| LeftThalamusProper | Leftparsopercularis | 0.798998 | Anterior limb of internal capsule |
| Rightsuperiorfrontal | Leftsuperiorfrontal | 0.795667 | Genu of corpus callosum |
| Leftsuperiorfrontal | Rightsuperiorfrontal | 0.795667 | Genu of corpus callosum |
| Rightprecentral | Leftprecentral | 0.791181 | Genu of corpus callosum |
| Leftprecentral | Rightprecentral | 0.791181 | Genu of corpus callosum |
| Leftmedialorbitofrontal | LeftPutamen | 0.789617 | Anterior corona radiata |
| LeftPutamen | Leftmedialorbitofrontal | 0.789617 | Anterior corona radiata |
| Rightsupramarginal | RightPutamen | 0.780691 | Fornix (cres) / Stria terminalis |
| RightPutamen | Rightsupramarginal | 0.780691 | Fornix (cres) / Stria terminalis |
| Rightsuperiorfrontal | Leftprecentral | 0.770302 | Genu of corpus callosum |
| Leftprecentral | Rightsuperiorfrontal | 0.770302 | Genu of corpus callosum |
| Leftisthmuscingulate | Leftprecuneus | 0.764454 | Body of corpus callosum |
| Leftprecuneus | Leftisthmuscingulate | 0.764454 | Body of corpus callosum |
| Leftsuperiorfrontal | Leftcaudalanteriorcingulate | 0.762295 | Cingulum (cingulate gyrus) |
| Leftcaudalanteriorcingulate | Leftsuperiorfrontal | 0.762295 | Cingulum (cingulate gyrus) |
| Rightcaudalanteriorcingulate | Leftrostralanteriorcingulate | 0.753513 | Cingulum (cingulate gyrus) |
| Leftrostralanteriorcingulate | Rightcaudalanteriorcingulate | 0.753513 | Cingulum (cingulate gyrus) |
| Rightsuperiorfrontal | RightThalamusProper | 0.752565 | Anterior corona radiata |
| RightThalamusProper | Rightsuperiorfrontal | 0.752565 | Anterior corona radiata |
| Rightparsorbitalis | RightThalamusProper | 0.751922 | Cerebral peduncle |
| RightThalamusProper | Rightparsorbitalis | 0.751922 | Cerebral peduncle |
| Rightrostralmiddlefrontal | RightThalamusProper | 0.751645 | Cerebral peduncle |
| RightThalamusProper | Rightrostralmiddlefrontal | 0.751645 | Cerebral peduncle |
| Rightprecentral | RightPutamen | 0.749784 | Anterior corona radiata |
| RightPutamen | Rightprecentral | 0.749784 | Anterior corona radiata |
| Leftparstriangularis | LeftCaudate | 0.744273 | Anterior corona radiata |
| LeftCaudate | Leftparstriangularis | 0.744273 | Anterior corona radiata |
| Rightinferiorparietal | Rightinferiortemporal | 0.741311 | Fornix (cres) / Stria terminalis |
| Rightinferiortemporal | Rightinferiorparietal | 0.741311 | Fornix (cres) / Stria terminalis |
| Rightcaudalmiddlefrontal | RightPutamen | 0.741165 | Anterior corona radiata |
| RightPutamen | Rightcaudalmiddlefrontal | 0.741165 | Anterior corona radiata |
| Rightprecentral | Leftparacentral | 0.740646 | Genu of corpus callosum |
| Leftparacentral | Rightprecentral | 0.740646 | Genu of corpus callosum |
| Rightinferiorparietal | RightPutamen | 0.73431 | Fornix (cres) / Stria terminalis |
| RightPutamen | Rightinferiorparietal | 0.73431 | Fornix (cres) / Stria terminalis |
| Leftrostralanteriorcingulate | Leftisthmuscingulate | 0.730102 | Cingulum (cingulate gyrus) |
| Leftisthmuscingulate | Leftrostralanteriorcingulate | 0.730102 | Cingulum (cingulate gyrus) |
| Leftparsorbitalis | LeftCaudate | 0.72928 | Anterior corona radiata |
| LeftCaudate | Leftparsorbitalis | 0.72928 | Anterior corona radiata |
| Rightpostcentral | Rightinsula | 0.727584 | Fornix (cres) / Stria terminalis |
| Rightinsula | Rightpostcentral | 0.727584 | Fornix (cres) / Stria terminalis |
| RightHippocampus | RightAmygdala | 0.721168 | Cingulum (hippocampus) |
| RightAmygdala | RightHippocampus | 0.721168 | Cingulum (hippocampus) |
| Rightsupramarginal | Rightmiddletemporal | 0.718525 | Fornix (cres) / Stria terminalis |
| Rightmiddletemporal | Rightsupramarginal | 0.718525 | Fornix (cres) / Stria terminalis |
| Rightprecentral | Rightsupramarginal | 0.716331 | Fornix (cres) / Stria terminalis |
| Rightsupramarginal | Rightprecentral | 0.716331 | Fornix (cres) / Stria terminalis |
| Leftisthmuscingulate | Leftcuneus | 0.715273 | Body of corpus callosum |
| Leftcuneus | Leftisthmuscingulate | 0.715273 | Body of corpus callosum |
| Rightparsopercularis | Rightinferiorparietal | 0.712273 | Fornix (cres) / Stria terminalis |
| Rightinferiorparietal | Rightparsopercularis | 0.712273 | Fornix (cres) / Stria terminalis |
| Rightcaudalanteriorcingulate | RightThalamusProper | 0.701724 | Cerebral peduncle |
| RightThalamusProper | Rightcaudalanteriorcingulate | 0.701724 | Cerebral peduncle |
| Leftsuperiortemporal | Leftinsula | 0.695773 | External capsule |
| Leftinsula | Leftsuperiortemporal | 0.695773 | External capsule |
| RightPutamen | Leftsuperiortemporal | 0.693446 | Body of corpus callosum |
| Leftsuperiortemporal | RightPutamen | 0.693446 | Body of corpus callosum |
| Rightposteriorcingulate | Leftposteriorcingulate | 0.691858 | Genu of corpus callosum |
| Leftposteriorcingulate | Rightposteriorcingulate | 0.691858 | Genu of corpus callosum |
| Rightparacentral | Leftparacentral | 0.688733 | Genu of corpus callosum |
| Leftparacentral | Rightparacentral | 0.688733 | Genu of corpus callosum |
| Rightparacentral | Rightposteriorcingulate | 0.685092 | Anterior corona radiata |
| Rightposteriorcingulate | Rightparacentral | 0.685092 | Anterior corona radiata |
| Rightisthmuscingulate | Rightsuperiorparietal | 0.683834 | Body of corpus callosum |
| Rightsuperiorparietal | Rightisthmuscingulate | 0.683834 | Body of corpus callosum |
| Rightcaudalmiddlefrontal | Leftsuperiorfrontal | 0.680797 | Genu of corpus callosum |
| Leftsuperiorfrontal | Rightcaudalmiddlefrontal | 0.680797 | Genu of corpus callosum |
| Rightrostralmiddlefrontal | RightPallidum | 0.674443 | Cerebral peduncle |
| RightPallidum | Rightrostralmiddlefrontal | 0.674443 | Cerebral peduncle |
| Leftposteriorcingulate | Leftprecuneus | 0.673193 | Cingulum (cingulate gyrus) |
| Leftprecuneus | Leftposteriorcingulate | 0.673193 | Cingulum (cingulate gyrus) |
| Rightcaudalanteriorcingulate | Leftcaudalanteriorcingulate | 0.665875 | Cingulum (cingulate gyrus) |
| Leftcaudalanteriorcingulate | Rightcaudalanteriorcingulate | 0.665875 | Cingulum (cingulate gyrus) |
| Rightparacentral | BrainStem | 0.663515 | Anterior limb of internal capsule |
| BrainStem | Rightparacentral | 0.663515 | Anterior limb of internal capsule |
| Rightlateraloccipital | Leftlateraloccipital | 0.663242 | Body of corpus callosum |
| Leftlateraloccipital | Rightlateraloccipital | 0.663242 | Body of corpus callosum |
| Leftparsorbitalis | LeftPutamen | 0.661987 | Anterior corona radiata |
| LeftPutamen | Leftparsorbitalis | 0.661987 | Anterior corona radiata |
| RightPutamen | RightPallidum | 0.657753 | Cerebral peduncle |
| RightPallidum | RightPutamen | 0.657753 | Cerebral peduncle |
| Rightparstriangularis | RightThalamusProper | 0.654981 | Cerebral peduncle |
| RightThalamusProper | Rightparstriangularis | 0.654981 | Cerebral peduncle |
| Rightprecuneus | Rightparahippocampal | 0.652113 | Body of corpus callosum |
| Rightparahippocampal | Rightprecuneus | 0.652113 | Body of corpus callosum |
| Leftparstriangularis | LeftPutamen | 0.650146 | Anterior corona radiata |
| LeftPutamen | Leftparstriangularis | 0.650146 | Anterior corona radiata |
| RightPallidum | LeftThalamusProper | 0.646457 | Anterior corona radiata |
| LeftThalamusProper | RightPallidum | 0.646457 | Anterior corona radiata |
| Rightmiddletemporal | BrainStem | 0.63812 | Anterior limb of internal capsule |
| BrainStem | Rightmiddletemporal | 0.63812 | Anterior limb of internal capsule |
| Rightprecentral | Rightinsula | 0.637897 | Fornix (cres) / Stria terminalis |
| Rightinsula | Rightprecentral | 0.637897 | Fornix (cres) / Stria terminalis |
| Rightprecuneus | Rightmiddletemporal | 0.635188 | Fornix (cres) / Stria terminalis |
| Rightmiddletemporal | Rightprecuneus | 0.635188 | Fornix (cres) / Stria terminalis |
| Rightparacentral | RightThalamusProper | 0.632404 | Anterior corona radiata |
| RightThalamusProper | Rightparacentral | 0.632404 | Anterior corona radiata |
| Rightsuperiorparietal | Rightbankssts | 0.617316 | Fornix (cres) / Stria terminalis |
| Rightbankssts | Rightsuperiorparietal | 0.617316 | Fornix (cres) / Stria terminalis |
| Rightisthmuscingulate | Rightlingual | 0.61425 | Cingulum (cingulate gyrus) |
| Rightlingual | Rightisthmuscingulate | 0.61425 | Cingulum (cingulate gyrus) |
| Rightsuperiorparietal | Leftpostcentral | 0.611007 | Genu of corpus callosum |
| Leftpostcentral | Rightsuperiorparietal | 0.611007 | Genu of corpus callosum |
| Rightsuperiorfrontal | Rightcaudalanteriorcingulate | 0.608073 | External capsule |
| Rightcaudalanteriorcingulate | Rightsuperiorfrontal | 0.608073 | External capsule |
| Rightisthmuscingulate | Leftisthmuscingulate | 0.606742 | Body of corpus callosum |
| Leftisthmuscingulate | Rightisthmuscingulate | 0.606742 | Body of corpus callosum |
| LeftThalamusProper | LeftCaudate | 0.60507 | Anterior limb of internal capsule |
| LeftCaudate | LeftThalamusProper | 0.60507 | Anterior limb of internal capsule |
| Rightparsopercularis | Rightsupramarginal | 0.604165 | Fornix (cres) / Stria terminalis |
| Rightsupramarginal | Rightparsopercularis | 0.604165 | Fornix (cres) / Stria terminalis |
| RightPutamen | Leftinsula | 0.60402 | Body of corpus callosum |
| Leftinsula | RightPutamen | 0.60402 | Body of corpus callosum |
| Rightposteriorcingulate | Leftisthmuscingulate | 0.595241 | Genu of corpus callosum |
| Leftisthmuscingulate | Rightposteriorcingulate | 0.595241 | Genu of corpus callosum |
| Leftsuperiortemporal | LeftPutamen | 0.59176 | External capsule |
| LeftPutamen | Leftsuperiortemporal | 0.59176 | External capsule |
| Rightprecuneus | BrainStem | 0.588933 | Anterior limb of internal capsule |
| BrainStem | Rightprecuneus | 0.588933 | Anterior limb of internal capsule |
| Rightprecuneus | Leftprecuneus | 0.585825 | Body of corpus callosum |
| Leftprecuneus | Rightprecuneus | 0.585825 | Body of corpus callosum |
| RightPutamen | LeftPallidum | 0.58557 | Genu of corpus callosum |
| LeftPallidum | RightPutamen | 0.58557 | Genu of corpus callosum |
| Leftcaudalmiddlefrontal | LeftPutamen | 0.584453 | External capsule |
| LeftPutamen | Leftcaudalmiddlefrontal | 0.584453 | External capsule |
| Rightisthmuscingulate | Rightprecuneus | 0.57857 | Body of corpus callosum |
| Rightprecuneus | Rightisthmuscingulate | 0.57857 | Body of corpus callosum |
| Rightprecentral | BrainStem | 0.577102 | Anterior limb of internal capsule |
| BrainStem | Rightprecentral | 0.577102 | Anterior limb of internal capsule |
| Rightparsopercularis | Rightbankssts | 0.570905 | Fornix (cres) / Stria terminalis |
| Rightbankssts | Rightparsopercularis | 0.570905 | Fornix (cres) / Stria terminalis |
| Rightposteriorcingulate | Rightprecuneus | 0.562347 | External capsule |
| Rightprecuneus | Rightposteriorcingulate | 0.562347 | External capsule |
| Rightparstriangularis | Rightprecentral | 0.557348 | Fornix (cres) / Stria terminalis |
| Rightprecentral | Rightparstriangularis | 0.557348 | Fornix (cres) / Stria terminalis |
| Leftinferiortemporal | LeftThalamusProper | 0.556817 | External capsule |
| LeftThalamusProper | Leftinferiortemporal | 0.556817 | External capsule |
| Rightparacentral | Leftpostcentral | 0.555877 | Genu of corpus callosum |
| Leftpostcentral | Rightparacentral | 0.555877 | Genu of corpus callosum |
| Leftlateralorbitofrontal | Leftmiddletemporal | 0.553177 | External capsule |
| Leftmiddletemporal | Leftlateralorbitofrontal | 0.553177 | External capsule |
| Leftisthmuscingulate | Leftparahippocampal | 0.552282 | Cingulum (hippocampus) |
| Leftparahippocampal | Leftisthmuscingulate | 0.552282 | Cingulum (hippocampus) |
| Leftprecuneus | Leftparahippocampal | 0.550474 | Body of corpus callosum |
| Leftparahippocampal | Leftprecuneus | 0.550474 | Body of corpus callosum |
| Rightsuperiorfrontal | BrainStem | 0.546827 | Anterior limb of internal capsule |
| BrainStem | Rightsuperiorfrontal | 0.546827 | Anterior limb of internal capsule |
| RightThalamusProper | Leftsuperiorparietal | 0.546184 | Body of corpus callosum |
| Leftsuperiorparietal | RightThalamusProper | 0.546184 | Body of corpus callosum |
| Rightpostcentral | BrainStem | 0.545513 | Anterior limb of internal capsule |
| BrainStem | Rightpostcentral | 0.545513 | Anterior limb of internal capsule |
| Leftsuperiorfrontal | Leftprecuneus | 0.543775 | Cingulum (cingulate gyrus) |
| Leftprecuneus | Leftsuperiorfrontal | 0.543775 | Cingulum (cingulate gyrus) |
| Rightposteriorcingulate | Leftprecuneus | 0.542324 | Cingulum (cingulate gyrus) |
| Leftprecuneus | Rightposteriorcingulate | 0.542324 | Cingulum (cingulate gyrus) |
| LeftPallidum | LeftHippocampus | 0.538696 | Cerebral peduncle |
| LeftHippocampus | LeftPallidum | 0.538696 | Cerebral peduncle |
| Rightcaudalanteriorcingulate | Rightposteriorcingulate | 0.538469 | External capsule |
| Rightposteriorcingulate | Rightcaudalanteriorcingulate | 0.538469 | External capsule |
| Leftrostralmiddlefrontal | LeftThalamusProper | 0.535843 | Anterior limb of internal capsule |
| LeftThalamusProper | Leftrostralmiddlefrontal | 0.535843 | Anterior limb of internal capsule |
| Rightprecuneus | Leftparacentral | 0.533517 | Cingulum (cingulate gyrus) |
| Leftparacentral | Rightprecuneus | 0.533517 | Cingulum (cingulate gyrus) |
| Rightparstriangularis | BrainStem | 0.532536 | Cerebral peduncle |
| BrainStem | Rightparstriangularis | 0.532536 | Cerebral peduncle |
| Rightprecentral | RightCaudate | 0.51952 | Anterior corona radiata |
| RightCaudate | Rightprecentral | 0.51952 | Anterior corona radiata |
| Leftrostralmiddlefrontal | LeftCaudate | 0.517517 | Anterior corona radiata |
| LeftCaudate | Leftrostralmiddlefrontal | 0.517517 | Anterior corona radiata |
| ***Probabilistic Tractograhy (Same Scanner)*** | | | |
| Rightlingual | Leftsuperiorparietal | 0.982547 | Body of corpus callosum |
| Leftsuperiorparietal | Rightlingual | 0.982547 | Body of corpus callosum |
| Rightcaudalmiddlefrontal | RightCaudate | 0.979497 | Anterior corona radiata |
| RightCaudate | Rightcaudalmiddlefrontal | 0.979497 | Anterior corona radiata |
| Rightparsopercularis | LeftPutamen | 0.968351 | Genu of corpus callosum |
| LeftPutamen | Rightparsopercularis | 0.968351 | Genu of corpus callosum |
| Rightcaudalmiddlefrontal | RightPutamen | 0.946578 | Anterior corona radiata |
| RightPutamen | Rightcaudalmiddlefrontal | 0.946578 | Anterior corona radiata |
| Leftlingual | Leftparahippocampal | 0.940642 | Cingulum (hippocampus) |
| Leftparahippocampal | Leftlingual | 0.940642 | Cingulum (hippocampus) |
| Rightsuperiorfrontal | LeftPallidum | 0.926775 | Genu of corpus callosum |
| LeftPallidum | Rightsuperiorfrontal | 0.926775 | Genu of corpus callosum |
| Rightprecuneus | Rightmiddletemporal | 0.925459 | Fornix (cres) / Stria terminalis |
| Rightmiddletemporal | Rightprecuneus | 0.925459 | Fornix (cres) / Stria terminalis |
| Leftparsopercularis | LeftPallidum | 0.920199 | Anterior corona radiata |
| LeftPallidum | Leftparsopercularis | 0.920199 | Anterior corona radiata |
| Rightlateraloccipital | Leftlateraloccipital | 0.915345 | Body of corpus callosum |
| Leftlateraloccipital | Rightlateraloccipital | 0.915345 | Body of corpus callosum |
| RightThalamusProper | LeftPutamen | 0.914903 | Anterior limb of internal capsule |
| LeftPutamen | RightThalamusProper | 0.914903 | Anterior limb of internal capsule |
| Rightsuperiorparietal | Leftsuperiorparietal | 0.913616 | Body of corpus callosum |
| Leftsuperiorparietal | Rightsuperiorparietal | 0.913616 | Body of corpus callosum |
| Rightcaudalanteriorcingulate | LeftPallidum | 0.9102 | Anterior corona radiata |
| LeftPallidum | Rightcaudalanteriorcingulate | 0.9102 | Anterior corona radiata |
| Rightparsopercularis | LeftThalamusProper | 0.909447 | Anterior corona radiata |
| LeftThalamusProper | Rightparsopercularis | 0.909447 | Anterior corona radiata |
| Rightsuperiorfrontal | RightPutamen | 0.908109 | Anterior corona radiata |
| RightPutamen | Rightsuperiorfrontal | 0.908109 | Anterior corona radiata |
| RightPutamen | LeftPutamen | 0.907658 | Anterior corona radiata |
| LeftPutamen | RightPutamen | 0.907658 | Anterior corona radiata |
| Rightprecuneus | Leftmedialorbitofrontal | 0.906297 | External capsule |
| Leftmedialorbitofrontal | Rightprecuneus | 0.906297 | External capsule |
| Rightprecentral | RightCaudate | 0.905536 | Anterior corona radiata |
| RightCaudate | Rightprecentral | 0.905536 | Anterior corona radiata |
| RightPutamen | Leftsuperiortemporal | 0.905191 | Body of corpus callosum |
| Leftsuperiortemporal | RightPutamen | 0.905191 | Body of corpus callosum |
| RightCaudate | LeftThalamusProper | 0.903391 | Genu of corpus callosum |
| LeftThalamusProper | RightCaudate | 0.903391 | Genu of corpus callosum |
| Rightpostcentral | Rightmiddletemporal | 0.897798 | Fornix (cres) / Stria terminalis |
| Rightmiddletemporal | Rightpostcentral | 0.897798 | Fornix (cres) / Stria terminalis |
| Leftparsopercularis | LeftPutamen | 0.896621 | Anterior corona radiata |
| LeftPutamen | Leftparsopercularis | 0.896621 | Anterior corona radiata |
| Rightisthmuscingulate | Rightsuperiorparietal | 0.895103 | Body of corpus callosum |
| Rightsuperiorparietal | Rightisthmuscingulate | 0.895103 | Body of corpus callosum |
| Leftparsopercularis | LeftCaudate | 0.895027 | Anterior corona radiata |
| LeftCaudate | Leftparsopercularis | 0.895027 | Anterior corona radiata |
| Rightisthmuscingulate | Leftisthmuscingulate | 0.894525 | Body of corpus callosum |
| Leftisthmuscingulate | Rightisthmuscingulate | 0.894525 | Body of corpus callosum |
| Rightsuperiorfrontal | LeftCaudate | 0.888448 | Genu of corpus callosum |
| LeftCaudate | Rightsuperiorfrontal | 0.888448 | Genu of corpus callosum |
| Rightsuperiorfrontal | RightThalamusProper | 0.886362 | Anterior corona radiata |
| RightThalamusProper | Rightsuperiorfrontal | 0.886362 | Anterior corona radiata |
| Leftparstriangularis | LeftCaudate | 0.886109 | Anterior corona radiata |
| LeftCaudate | Leftparstriangularis | 0.886109 | Anterior corona radiata |
| RightPallidum | LeftThalamusProper | 0.885962 | Anterior corona radiata |
| LeftThalamusProper | RightPallidum | 0.885962 | Anterior corona radiata |
| Leftisthmuscingulate | Leftsuperiorparietal | 0.885213 | Body of corpus callosum |
| Leftsuperiorparietal | Leftisthmuscingulate | 0.885213 | Body of corpus callosum |
| Rightcaudalmiddlefrontal | Rightmiddletemporal | 0.881242 | Fornix (cres) / Stria terminalis |
| Rightmiddletemporal | Rightcaudalmiddlefrontal | 0.881242 | Fornix (cres) / Stria terminalis |
| RightPutamen | Leftisthmuscingulate | 0.877095 | Fornix (cres) / Stria terminalis |
| Leftisthmuscingulate | RightPutamen | 0.877095 | Fornix (cres) / Stria terminalis |
| Rightrostralmiddlefrontal | Rightinferiorparietal | 0.870747 | Fornix (cres) / Stria terminalis |
| Rightinferiorparietal | Rightrostralmiddlefrontal | 0.870747 | Fornix (cres) / Stria terminalis |
| Rightcuneus | Leftpericalcarine | 0.868192 | Body of corpus callosum |
| Leftpericalcarine | Rightcuneus | 0.868192 | Body of corpus callosum |
| RightPutamen | Leftinferiortemporal | 0.864515 | Body of corpus callosum |
| Leftinferiortemporal | RightPutamen | 0.864515 | Body of corpus callosum |
| LeftCaudate | LeftPutamen | 0.862409 | Anterior limb of internal capsule |
| LeftPutamen | LeftCaudate | 0.862409 | Anterior limb of internal capsule |
| Rightprecuneus | Rightbankssts | 0.859386 | Fornix (cres) / Stria terminalis |
| Rightbankssts | Rightprecuneus | 0.859386 | Fornix (cres) / Stria terminalis |
| Rightcaudalmiddlefrontal | RightPallidum | 0.857249 | Cerebral peduncle |
| RightPallidum | Rightcaudalmiddlefrontal | 0.857249 | Cerebral peduncle |
| RightThalamusProper | Leftsuperiorfrontal | 0.856811 | Genu of corpus callosum |
| Leftsuperiorfrontal | RightThalamusProper | 0.856811 | Genu of corpus callosum |
| Rightrostralmiddlefrontal | LeftThalamusProper | 0.856436 | Anterior corona radiata |
| LeftThalamusProper | Rightrostralmiddlefrontal | 0.856436 | Anterior corona radiata |
| Rightprecentral | Rightbankssts | 0.853471 | Fornix (cres) / Stria terminalis |
| Rightbankssts | Rightprecentral | 0.853471 | Fornix (cres) / Stria terminalis |
| Rightcaudalmiddlefrontal | RightThalamusProper | 0.851541 | Anterior corona radiata |
| RightThalamusProper | Rightcaudalmiddlefrontal | 0.851541 | Anterior corona radiata |
| Leftrostralanteriorcingulate | Leftisthmuscingulate | 0.850258 | Cingulum (cingulate gyrus) |
| Leftisthmuscingulate | Leftrostralanteriorcingulate | 0.850258 | Cingulum (cingulate gyrus) |
| Rightsuperiorfrontal | RightPallidum | 0.849742 | Anterior corona radiata |
| RightPallidum | Rightsuperiorfrontal | 0.849742 | Anterior corona radiata |
| Rightinferiorparietal | Rightsuperiortemporal | 0.849505 | Fornix (cres) / Stria terminalis |
| Rightsuperiortemporal | Rightinferiorparietal | 0.849505 | Fornix (cres) / Stria terminalis |
| Rightisthmuscingulate | Leftsuperiorparietal | 0.849299 | Body of corpus callosum |
| Leftsuperiorparietal | Rightisthmuscingulate | 0.849299 | Body of corpus callosum |
| Leftlateralorbitofrontal | Leftinferiorparietal | 0.8433 | External capsule |
| Leftinferiorparietal | Leftlateralorbitofrontal | 0.8433 | External capsule |
| Rightsuperiorfrontal | Leftprecentral | 0.841653 | Genu of corpus callosum |
| Leftprecentral | Rightsuperiorfrontal | 0.841653 | Genu of corpus callosum |
| Rightprecentral | Rightmiddletemporal | 0.841651 | Fornix (cres) / Stria terminalis |
| Rightmiddletemporal | Rightprecentral | 0.841651 | Fornix (cres) / Stria terminalis |
| Rightprecuneus | BrainStem | 0.839426 | Anterior limb of internal capsule |
| BrainStem | Rightprecuneus | 0.839426 | Anterior limb of internal capsule |
| Rightrostralmiddlefrontal | RightPallidum | 0.834849 | Cerebral peduncle |
| RightPallidum | Rightrostralmiddlefrontal | 0.834849 | Cerebral peduncle |
| Rightprecuneus | Leftsuperiorparietal | 0.834007 | Body of corpus callosum |
| Leftsuperiorparietal | Rightprecuneus | 0.834007 | Body of corpus callosum |
| Rightparstriangularis | RightThalamusProper | 0.833989 | Cerebral peduncle |
| RightThalamusProper | Rightparstriangularis | 0.833989 | Cerebral peduncle |
| Leftrostralmiddlefrontal | LeftThalamusProper | 0.833411 | Anterior limb of internal capsule |
| LeftThalamusProper | Leftrostralmiddlefrontal | 0.833411 | Anterior limb of internal capsule |
| RightPutamen | Leftprecuneus | 0.833012 | Body of corpus callosum |
| Leftprecuneus | RightPutamen | 0.833012 | Body of corpus callosum |
| Leftinferiortemporal | LeftPutamen | 0.832549 | External capsule |
| LeftPutamen | Leftinferiortemporal | 0.832549 | External capsule |
| Leftcaudalanteriorcingulate | LeftAccumbensarea | 0.831273 | Anterior corona radiata |
| LeftAccumbensarea | Leftcaudalanteriorcingulate | 0.831273 | Anterior corona radiata |
| Rightsuperiorfrontal | LeftPutamen | 0.831196 | Genu of corpus callosum |
| LeftPutamen | Rightsuperiorfrontal | 0.831196 | Genu of corpus callosum |
| Leftisthmuscingulate | Leftsuperiortemporal | 0.830035 | Body of corpus callosum |
| Leftsuperiortemporal | Leftisthmuscingulate | 0.830035 | Body of corpus callosum |
| RightPutamen | Leftlateraloccipital | 0.82882 | Body of corpus callosum |
| Leftlateraloccipital | RightPutamen | 0.82882 | Body of corpus callosum |
| Rightprecentral | LeftThalamusProper | 0.827342 | Genu of corpus callosum |
| LeftThalamusProper | Rightprecentral | 0.827342 | Genu of corpus callosum |
| Rightsuperiorfrontal | Rightisthmuscingulate | 0.825905 | External capsule |
| Rightisthmuscingulate | Rightsuperiorfrontal | 0.825905 | External capsule |
| Rightcaudalmiddlefrontal | Rightinferiorparietal | 0.825591 | Fornix (cres) / Stria terminalis |
| Rightinferiorparietal | Rightcaudalmiddlefrontal | 0.825591 | Fornix (cres) / Stria terminalis |
| Rightcaudalanteriorcingulate | Leftcaudalanteriorcingulate | 0.825478 | Cingulum (cingulate gyrus) |
| Leftcaudalanteriorcingulate | Rightcaudalanteriorcingulate | 0.825478 | Cingulum (cingulate gyrus) |
| Rightsuperiorparietal | Leftisthmuscingulate | 0.824739 | Body of corpus callosum |
| Leftisthmuscingulate | Rightsuperiorparietal | 0.824739 | Body of corpus callosum |
| Leftcaudalmiddlefrontal | LeftPutamen | 0.824472 | External capsule |
| LeftPutamen | Leftcaudalmiddlefrontal | 0.824472 | External capsule |
| Leftparstriangularis | LeftPutamen | 0.824115 | Anterior corona radiata |
| LeftPutamen | Leftparstriangularis | 0.824115 | Anterior corona radiata |
| Rightpericalcarine | Leftsuperiorparietal | 0.823886 | Body of corpus callosum |
| Leftsuperiorparietal | Rightpericalcarine | 0.823886 | Body of corpus callosum |
| Leftrostralmiddlefrontal | LeftPutamen | 0.821463 | Anterior corona radiata |
| LeftPutamen | Leftrostralmiddlefrontal | 0.821463 | Anterior corona radiata |
| Rightprecuneus | Rightlingual | 0.819765 | Body of corpus callosum |
| Rightlingual | Rightprecuneus | 0.819765 | Body of corpus callosum |
| Rightprecuneus | Leftisthmuscingulate | 0.818867 | Body of corpus callosum |
| Leftisthmuscingulate | Rightprecuneus | 0.818867 | Body of corpus callosum |
| Leftfrontalpole | LeftCaudate | 0.816685 | Anterior corona radiata |
| LeftCaudate | Leftfrontalpole | 0.816685 | Anterior corona radiata |
| Rightlateraloccipital | Leftcuneus | 0.81532 | Body of corpus callosum |
| Leftcuneus | Rightlateraloccipital | 0.81532 | Body of corpus callosum |
| Leftrostralmiddlefrontal | LeftCaudate | 0.814201 | Anterior corona radiata |
| LeftCaudate | Leftrostralmiddlefrontal | 0.814201 | Anterior corona radiata |
| Rightisthmuscingulate | Rightprecuneus | 0.813193 | Body of corpus callosum |
| Rightprecuneus | Rightisthmuscingulate | 0.813193 | Body of corpus callosum |
| Rightsuperiortemporal | LeftThalamusProper | 0.810268 | Body of corpus callosum |
| LeftThalamusProper | Rightsuperiortemporal | 0.810268 | Body of corpus callosum |
| Rightsuperiorfrontal | Leftsuperiorfrontal | 0.806341 | Genu of corpus callosum |
| Leftsuperiorfrontal | Rightsuperiorfrontal | 0.806341 | Genu of corpus callosum |
| Leftfrontalpole | LeftPutamen | 0.804672 | Anterior corona radiata |
| LeftPutamen | Leftfrontalpole | 0.804672 | Anterior corona radiata |
| Rightcaudalmiddlefrontal | Rightbankssts | 0.804481 | Fornix (cres) / Stria terminalis |
| Rightbankssts | Rightcaudalmiddlefrontal | 0.804481 | Fornix (cres) / Stria terminalis |
| Rightcaudalanteriorcingulate | Leftrostralmiddlefrontal | 0.804414 | Anterior corona radiata |
| Leftrostralmiddlefrontal | Rightcaudalanteriorcingulate | 0.804414 | Anterior corona radiata |
| Rightinferiorparietal | RightPutamen | 0.803956 | Fornix (cres) / Stria terminalis |
| RightPutamen | Rightinferiorparietal | 0.803956 | Fornix (cres) / Stria terminalis |
| Rightsuperiorfrontal | Leftparacentral | 0.80084 | Genu of corpus callosum |
| Leftparacentral | Rightsuperiorfrontal | 0.80084 | Genu of corpus callosum |
| Rightisthmuscingulate | Rightpericalcarine | 0.795947 | Cingulum (cingulate gyrus) |
| Rightpericalcarine | Rightisthmuscingulate | 0.795947 | Cingulum (cingulate gyrus) |
| Rightrostralmiddlefrontal | Rightmiddletemporal | 0.79588 | Fornix (cres) / Stria terminalis |
| Rightmiddletemporal | Rightrostralmiddlefrontal | 0.79588 | Fornix (cres) / Stria terminalis |
| Rightinsula | Leftisthmuscingulate | 0.793877 | Body of corpus callosum |
| Leftisthmuscingulate | Rightinsula | 0.793877 | Body of corpus callosum |
| Rightcaudalanteriorcingulate | Leftsuperiorfrontal | 0.793876 | Cingulum (cingulate gyrus) |
| Leftsuperiorfrontal | Rightcaudalanteriorcingulate | 0.793876 | Cingulum (cingulate gyrus) |
| Rightprecuneus | LeftPutamen | 0.793591 | Body of corpus callosum |
| LeftPutamen | Rightprecuneus | 0.793591 | Body of corpus callosum |
| RightPutamen | Leftinsula | 0.791106 | Body of corpus callosum |
| Leftinsula | RightPutamen | 0.791106 | Body of corpus callosum |
| Leftparsorbitalis | LeftCaudate | 0.790991 | Anterior corona radiata |
| LeftCaudate | Leftparsorbitalis | 0.790991 | Anterior corona radiata |
| Rightmiddletemporal | RightPutamen | 0.789255 | Fornix (cres) / Stria terminalis |
| RightPutamen | Rightmiddletemporal | 0.789255 | Fornix (cres) / Stria terminalis |
| RightThalamusProper | RightPutamen | 0.785413 | Anterior limb of internal capsule |
| RightPutamen | RightThalamusProper | 0.785413 | Anterior limb of internal capsule |
| Rightsuperiorparietal | Rightbankssts | 0.785017 | Fornix (cres) / Stria terminalis |
| Rightbankssts | Rightsuperiorparietal | 0.785017 | Fornix (cres) / Stria terminalis |
| RightThalamusProper | RightPallidum | 0.783986 | Anterior limb of internal capsule |
| RightPallidum | RightThalamusProper | 0.783986 | Anterior limb of internal capsule |
| Rightprecentral | BrainStem | 0.783408 | Anterior limb of internal capsule |
| BrainStem | Rightprecentral | 0.783408 | Anterior limb of internal capsule |
| Rightrostralmiddlefrontal | LeftPutamen | 0.78331 | Anterior corona radiata |
| LeftPutamen | Rightrostralmiddlefrontal | 0.78331 | Anterior corona radiata |
| Leftcaudalanteriorcingulate | LeftCaudate | 0.782735 | Anterior corona radiata |
| LeftCaudate | Leftcaudalanteriorcingulate | 0.782735 | Anterior corona radiata |
| RightThalamusProper | Leftisthmuscingulate | 0.782479 | Body of corpus callosum |
| Leftisthmuscingulate | RightThalamusProper | 0.782479 | Body of corpus callosum |
| Rightrostralmiddlefrontal | LeftPallidum | 0.781981 | Anterior limb of internal capsule |
| LeftPallidum | Rightrostralmiddlefrontal | 0.781981 | Anterior limb of internal capsule |
| Rightrostralmiddlefrontal | Rightcaudalanteriorcingulate | 0.781447 | External capsule |
| Rightcaudalanteriorcingulate | Rightrostralmiddlefrontal | 0.781447 | External capsule |
| Rightsuperiorparietal | Leftinferiorparietal | 0.780107 | Body of corpus callosum |
| Leftinferiorparietal | Rightsuperiorparietal | 0.780107 | Body of corpus callosum |
| Rightinferiorparietal | Righttransversetemporal | 0.779296 | Fornix (cres) / Stria terminalis |
| Righttransversetemporal | Rightinferiorparietal | 0.779296 | Fornix (cres) / Stria terminalis |
| ***Probabilistic Tractograhy Different Scanners)*** | | | |
| Gray matter region | Gray matter region | ICC | White matter region |
| RightCaudate | LeftThalamusProper | 0.958837 | Genu of corpus callosum |
| LeftThalamusProper | RightCaudate | 0.958837 | Genu of corpus callosum |
| Rightpericalcarine | Leftsuperiorparietal | 0.915874 | Body of corpus callosum |
| Leftsuperiorparietal | Rightpericalcarine | 0.915874 | Body of corpus callosum |
| Rightprecentral | RightCaudate | 0.908125 | Anterior corona radiata |
| RightCaudate | Rightprecentral | 0.908125 | Anterior corona radiata |
| Rightisthmuscingulate | Rightpericalcarine | 0.876481 | Cingulum (cingulate gyrus) |
| Rightpericalcarine | Rightisthmuscingulate | 0.876481 | Cingulum (cingulate gyrus) |
| Rightparsopercularis | LeftPutamen | 0.872963 | Genu of corpus callosum |
| LeftPutamen | Rightparsopercularis | 0.872963 | Genu of corpus callosum |
| Rightpostcentral | Leftsuperiorparietal | 0.871714 | Genu of corpus callosum |
| Leftsuperiorparietal | Rightpostcentral | 0.871714 | Genu of corpus callosum |
| Leftparsopercularis | LeftCaudate | 0.869968 | Anterior corona radiata |
| LeftCaudate | Leftparsopercularis | 0.869968 | Anterior corona radiata |
| Rightlateraloccipital | Leftlateraloccipital | 0.869576 | Body of corpus callosum |
| Leftlateraloccipital | Rightlateraloccipital | 0.869576 | Body of corpus callosum |
| Rightsuperiortemporal | LeftPutamen | 0.864824 | Body of corpus callosum |
| LeftPutamen | Rightsuperiortemporal | 0.864824 | Body of corpus callosum |
| RightThalamusProper | Leftsuperiorfrontal | 0.855248 | Genu of corpus callosum |
| Leftsuperiorfrontal | RightThalamusProper | 0.855248 | Genu of corpus callosum |
| Leftisthmuscingulate | Leftsuperiortemporal | 0.847681 | Body of corpus callosum |
| Leftsuperiortemporal | Leftisthmuscingulate | 0.847681 | Body of corpus callosum |
| Leftparsopercularis | LeftPutamen | 0.845874 | Anterior corona radiata |
| LeftPutamen | Leftparsopercularis | 0.845874 | Anterior corona radiata |
| Rightsupramarginal | Rightmiddletemporal | 0.840119 | Fornix (cres) / Stria terminalis |
| Rightmiddletemporal | Rightsupramarginal | 0.840119 | Fornix (cres) / Stria terminalis |
| Rightparsopercularis | Rightmiddletemporal | 0.835702 | Fornix (cres) / Stria terminalis |
| Rightmiddletemporal | Rightparsopercularis | 0.835702 | Fornix (cres) / Stria terminalis |
| Rightsuperiorparietal | Leftposteriorcingulate | 0.831356 | Genu of corpus callosum |
| Leftposteriorcingulate | Rightsuperiorparietal | 0.831356 | Genu of corpus callosum |
| Rightinsula | LeftPutamen | 0.83114 | Body of corpus callosum |
| LeftPutamen | Rightinsula | 0.83114 | Body of corpus callosum |
| Rightprecuneus | Rightbankssts | 0.829689 | Fornix (cres) / Stria terminalis |
| Rightbankssts | Rightprecuneus | 0.829689 | Fornix (cres) / Stria terminalis |
| RightPutamen | Leftinferiortemporal | 0.826688 | Body of corpus callosum |
| Leftinferiortemporal | RightPutamen | 0.826688 | Body of corpus callosum |
| Leftparsopercularis | LeftThalamusProper | 0.824658 | Anterior limb of internal capsule |
| LeftThalamusProper | Leftparsopercularis | 0.824658 | Anterior limb of internal capsule |
| Rightcaudalmiddlefrontal | Rightinferiorparietal | 0.822497 | Fornix (cres) / Stria terminalis |
| Rightinferiorparietal | Rightcaudalmiddlefrontal | 0.822497 | Fornix (cres) / Stria terminalis |
| Rightsuperiorfrontal | Leftparsopercularis | 0.82226 | Anterior corona radiata |
| Leftparsopercularis | Rightsuperiorfrontal | 0.82226 | Anterior corona radiata |
| Rightinsula | Leftisthmuscingulate | 0.820842 | Body of corpus callosum |
| Leftisthmuscingulate | Rightinsula | 0.820842 | Body of corpus callosum |
| Rightparsopercularis | LeftThalamusProper | 0.817728 | Anterior corona radiata |
| LeftThalamusProper | Rightparsopercularis | 0.817728 | Anterior corona radiata |
| Rightpericalcarine | Leftpericalcarine | 0.816543 | Body of corpus callosum |
| Leftpericalcarine | Rightpericalcarine | 0.816543 | Body of corpus callosum |
| RightPallidum | Leftsuperiorfrontal | 0.809453 | Genu of corpus callosum |
| Leftsuperiorfrontal | RightPallidum | 0.809453 | Genu of corpus callosum |
| Rightisthmuscingulate | Rightsuperiorparietal | 0.808449 | Body of corpus callosum |
| Rightsuperiorparietal | Rightisthmuscingulate | 0.808449 | Body of corpus callosum |
| Rightprecentral | Leftsuperiorfrontal | 0.808317 | Genu of corpus callosum |
| Leftsuperiorfrontal | Rightprecentral | 0.808317 | Genu of corpus callosum |
| Rightparstriangularis | RightThalamusProper | 0.804568 | Cerebral peduncle |
| RightThalamusProper | Rightparstriangularis | 0.804568 | Cerebral peduncle |
| Rightpostcentral | Rightmiddletemporal | 0.797129 | Fornix (cres) / Stria terminalis |
| Rightmiddletemporal | Rightpostcentral | 0.797129 | Fornix (cres) / Stria terminalis |
| Rightsuperiorfrontal | RightPallidum | 0.795694 | Anterior corona radiata |
| RightPallidum | Rightsuperiorfrontal | 0.795694 | Anterior corona radiata |
| Rightcaudalanteriorcingulate | Rightposteriorcingulate | 0.794719 | External capsule |
| Rightposteriorcingulate | Rightcaudalanteriorcingulate | 0.794719 | External capsule |
| Rightprecuneus | Rightmiddletemporal | 0.791301 | Fornix (cres) / Stria terminalis |
| Rightmiddletemporal | Rightprecuneus | 0.791301 | Fornix (cres) / Stria terminalis |
| Lefttemporalpole | LeftThalamusProper | 0.789389 | External capsule |
| LeftThalamusProper | Lefttemporalpole | 0.789389 | External capsule |
| Rightprecuneus | Leftlateraloccipital | 0.78881 | Body of corpus callosum |
| Leftlateraloccipital | Rightprecuneus | 0.78881 | Body of corpus callosum |
| Rightcaudalmiddlefrontal | RightThalamusProper | 0.785628 | Anterior corona radiata |
| RightThalamusProper | Rightcaudalmiddlefrontal | 0.785628 | Anterior corona radiata |
| Rightrostralmiddlefrontal | LeftPallidum | 0.780184 | Anterior limb of internal capsule |
| LeftPallidum | Rightrostralmiddlefrontal | 0.780184 | Anterior limb of internal capsule |
| Rightprecentral | RightHippocampus | 0.77899 | Anterior corona radiata |
| RightHippocampus | Rightprecentral | 0.77899 | Anterior corona radiata |
| Rightsuperiorfrontal | Leftprecentral | 0.765049 | Genu of corpus callosum |
| Leftprecentral | Rightsuperiorfrontal | 0.765049 | Genu of corpus callosum |
| Leftrostralmiddlefrontal | Leftcaudalanteriorcingulate | 0.763792 | Cingulum (cingulate gyrus) |
| Leftcaudalanteriorcingulate | Leftrostralmiddlefrontal | 0.763792 | Cingulum (cingulate gyrus) |
| Rightsuperiorparietal | Rightbankssts | 0.759323 | Fornix (cres) / Stria terminalis |
| Rightbankssts | Rightsuperiorparietal | 0.759323 | Fornix (cres) / Stria terminalis |
| RightPallidum | LeftThalamusProper | 0.753763 | Anterior corona radiata |
| LeftThalamusProper | RightPallidum | 0.753763 | Anterior corona radiata |
| Leftparsopercularis | LeftPallidum | 0.752891 | Anterior corona radiata |
| LeftPallidum | Leftparsopercularis | 0.752891 | Anterior corona radiata |
| Leftlingual | Leftparahippocampal | 0.749895 | Cingulum (hippocampus) |
| Leftparahippocampal | Leftlingual | 0.749895 | Cingulum (hippocampus) |
| Rightcuneus | Leftpericalcarine | 0.748824 | Body of corpus callosum |
| Leftpericalcarine | Rightcuneus | 0.748824 | Body of corpus callosum |
| Rightcaudalanteriorcingulate | Leftcaudalanteriorcingulate | 0.745711 | Cingulum (cingulate gyrus) |
| Leftcaudalanteriorcingulate | Rightcaudalanteriorcingulate | 0.745711 | Cingulum (cingulate gyrus) |
| Rightcaudalmiddlefrontal | RightPutamen | 0.742745 | Anterior corona radiata |
| RightPutamen | Rightcaudalmiddlefrontal | 0.742745 | Anterior corona radiata |
| Rightsuperiorfrontal | Leftcaudalmiddlefrontal | 0.740878 | Cingulum (cingulate gyrus) |
| Leftcaudalmiddlefrontal | Rightsuperiorfrontal | 0.740878 | Cingulum (cingulate gyrus) |
| RightPutamen | Leftsuperiortemporal | 0.736894 | Body of corpus callosum |
| Leftsuperiortemporal | RightPutamen | 0.736894 | Body of corpus callosum |
| Rightlateraloccipital | Leftpericalcarine | 0.735545 | Body of corpus callosum |
| Leftpericalcarine | Rightlateraloccipital | 0.735545 | Body of corpus callosum |
| Leftisthmuscingulate | Leftlateraloccipital | 0.735037 | Body of corpus callosum |
| Leftlateraloccipital | Leftisthmuscingulate | 0.735037 | Body of corpus callosum |
| Rightinsula | LeftThalamusProper | 0.733474 | Body of corpus callosum |
| LeftThalamusProper | Rightinsula | 0.733474 | Body of corpus callosum |
| Leftisthmuscingulate | LeftPutamen | 0.732212 | Body of corpus callosum |
| LeftPutamen | Leftisthmuscingulate | 0.732212 | Body of corpus callosum |
| Rightprecuneus | BrainStem | 0.732101 | Anterior limb of internal capsule |
| BrainStem | Rightprecuneus | 0.732101 | Anterior limb of internal capsule |
| Rightsupramarginal | Rightprecuneus | 0.730191 | Fornix (cres) / Stria terminalis |
| Rightprecuneus | Rightsupramarginal | 0.730191 | Fornix (cres) / Stria terminalis |
| Rightsuperiorfrontal | BrainStem | 0.726634 | Anterior limb of internal capsule |
| BrainStem | Rightsuperiorfrontal | 0.726634 | Anterior limb of internal capsule |
| Rightcuneus | Leftprecuneus | 0.726382 | Body of corpus callosum |
| Leftprecuneus | Rightcuneus | 0.726382 | Body of corpus callosum |
| Leftisthmuscingulate | Leftcuneus | 0.724961 | Body of corpus callosum |
| Leftcuneus | Leftisthmuscingulate | 0.724961 | Body of corpus callosum |
| Rightisthmuscingulate | Rightlingual | 0.724223 | Cingulum (cingulate gyrus) |
| Rightlingual | Rightisthmuscingulate | 0.724223 | Cingulum (cingulate gyrus) |
| Rightrostralmiddlefrontal | Rightcaudalanteriorcingulate | 0.723153 | External capsule |
| Rightcaudalanteriorcingulate | Rightrostralmiddlefrontal | 0.723153 | External capsule |
| Leftparstriangularis | LeftCaudate | 0.722921 | Anterior corona radiata |
| LeftCaudate | Leftparstriangularis | 0.722921 | Anterior corona radiata |
| Leftisthmuscingulate | Leftinferiorparietal | 0.719419 | Body of corpus callosum |
| Leftinferiorparietal | Leftisthmuscingulate | 0.719419 | Body of corpus callosum |
| Rightlateraloccipital | Leftsuperiorparietal | 0.716841 | Body of corpus callosum |
| Leftsuperiorparietal | Rightlateraloccipital | 0.716841 | Body of corpus callosum |
| RightThalamusProper | RightHippocampus | 0.713226 | Cingulum (hippocampus) |
| RightHippocampus | RightThalamusProper | 0.713226 | Cingulum (hippocampus) |
| RightThalamusProper | RightPutamen | 0.712695 | Anterior limb of internal capsule |
| RightPutamen | RightThalamusProper | 0.712695 | Anterior limb of internal capsule |
| Rightposteriorcingulate | Leftsuperiorparietal | 0.711064 | Genu of corpus callosum |
| Leftsuperiorparietal | Rightposteriorcingulate | 0.711064 | Genu of corpus callosum |
| Rightprecentral | Rightmiddletemporal | 0.709605 | Fornix (cres) / Stria terminalis |
| Rightmiddletemporal | Rightprecentral | 0.709605 | Fornix (cres) / Stria terminalis |
| Leftisthmuscingulate | Leftsuperiorparietal | 0.704274 | Body of corpus callosum |
| Leftsuperiorparietal | Leftisthmuscingulate | 0.704274 | Body of corpus callosum |
| RightThalamusProper | LeftPutamen | 0.703837 | Anterior limb of internal capsule |
| LeftPutamen | RightThalamusProper | 0.703837 | Anterior limb of internal capsule |
| Rightsuperiorparietal | Leftisthmuscingulate | 0.703674 | Body of corpus callosum |
| Leftisthmuscingulate | Rightsuperiorparietal | 0.703674 | Body of corpus callosum |
| Rightprecuneus | Rightparahippocampal | 0.70096 | Body of corpus callosum |
| Rightparahippocampal | Rightprecuneus | 0.70096 | Body of corpus callosum |
| Leftrostralanteriorcingulate | Leftisthmuscingulate | 0.700716 | Cingulum (cingulate gyrus) |
| Leftisthmuscingulate | Leftrostralanteriorcingulate | 0.700716 | Cingulum (cingulate gyrus) |
| RightThalamusProper | Leftisthmuscingulate | 0.69694 | Body of corpus callosum |
| Leftisthmuscingulate | RightThalamusProper | 0.69694 | Body of corpus callosum |
| LeftThalamusProper | LeftAmygdala | 0.694819 | Cerebral peduncle |
| LeftAmygdala | LeftThalamusProper | 0.694819 | Cerebral peduncle |
| Rightcaudalmiddlefrontal | RightCaudate | 0.694739 | Anterior corona radiata |
| RightCaudate | Rightcaudalmiddlefrontal | 0.694739 | Anterior corona radiata |
| Rightparsopercularis | Rightsupramarginal | 0.694424 | Fornix (cres) / Stria terminalis |
| Rightsupramarginal | Rightparsopercularis | 0.694424 | Fornix (cres) / Stria terminalis |
| Leftlateralorbitofrontal | Leftfusiform | 0.69305 | External capsule |
| Leftfusiform | Leftlateralorbitofrontal | 0.69305 | External capsule |
| Leftparahippocampal | LeftHippocampus | 0.692463 | Cingulum (hippocampus) |
| LeftHippocampus | Leftparahippocampal | 0.692463 | Cingulum (hippocampus) |
| Rightisthmuscingulate | Leftisthmuscingulate | 0.682658 | Body of corpus callosum |
| Leftisthmuscingulate | Rightisthmuscingulate | 0.682658 | Body of corpus callosum |
| RightPallidum | Leftcaudalmiddlefrontal | 0.68209 | Genu of corpus callosum |
| Leftcaudalmiddlefrontal | RightPallidum | 0.68209 | Genu of corpus callosum |
| Rightrostralmiddlefrontal | RightPallidum | 0.68039 | Cerebral peduncle |
| RightPallidum | Rightrostralmiddlefrontal | 0.68039 | Cerebral peduncle |
| RightHippocampus | RightAmygdala | 0.679894 | Cingulum (hippocampus) |
| RightAmygdala | RightHippocampus | 0.679894 | Cingulum (hippocampus) |
| Rightsuperiortemporal | Leftlingual | 0.679795 | Body of corpus callosum |
| Leftlingual | Rightsuperiortemporal | 0.679795 | Body of corpus callosum |
| Rightpostcentral | RightPutamen | 0.679417 | Anterior corona radiata |
| RightPutamen | Rightpostcentral | 0.679417 | Anterior corona radiata |
| Rightposteriorcingulate | Leftprecuneus | 0.677674 | Cingulum (cingulate gyrus) |
| Leftprecuneus | Rightposteriorcingulate | 0.677674 | Cingulum (cingulate gyrus) |
| Leftisthmuscingulate | Leftparahippocampal | 0.673084 | Cingulum (hippocampus) |
| Leftparahippocampal | Leftisthmuscingulate | 0.673084 | Cingulum (hippocampus) |
| RightThalamusProper | RightPallidum | 0.671468 | Anterior limb of internal capsule |
| RightPallidum | RightThalamusProper | 0.671468 | Anterior limb of internal capsule |
| Rightpericalcarine | Leftlateraloccipital | 0.665634 | Body of corpus callosum |
| Leftlateraloccipital | Rightpericalcarine | 0.665634 | Body of corpus callosum |
| Leftrostralmiddlefrontal | LeftPallidum | 0.66534 | Anterior corona radiata |
| LeftPallidum | Leftrostralmiddlefrontal | 0.66534 | Anterior corona radiata |
| Rightpericalcarine | Leftcuneus | 0.657387 | Body of corpus callosum |
| Leftcuneus | Rightpericalcarine | 0.657387 | Body of corpus callosum |
| Rightcaudalanteriorcingulate | LeftPallidum | 0.657177 | Anterior corona radiata |
| LeftPallidum | Rightcaudalanteriorcingulate | 0.657177 | Anterior corona radiata |
| Rightisthmuscingulate | Leftsuperiorparietal | 0.656432 | Body of corpus callosum |
| Leftsuperiorparietal | Rightisthmuscingulate | 0.656432 | Body of corpus callosum |
| Righttemporalpole | RightThalamusProper | 0.656256 | Cingulum (hippocampus) |
| RightThalamusProper | Righttemporalpole | 0.656256 | Cingulum (hippocampus) |
| Rightposteriorcingulate | Rightsuperiorparietal | 0.655166 | Anterior corona radiata |
| Rightsuperiorparietal | Rightposteriorcingulate | 0.655166 | Anterior corona radiata |
| Rightsuperiortemporal | LeftThalamusProper | 0.654608 | Body of corpus callosum |
| LeftThalamusProper | Rightsuperiortemporal | 0.654608 | Body of corpus callosum |
| Rightprecentral | Rightinferiortemporal | 0.653513 | Fornix (cres) / Stria terminalis |
| Rightinferiortemporal | Rightprecentral | 0.653513 | Fornix (cres) / Stria terminalis |
| Rightpostcentral | RightPallidum | 0.652646 | Anterior corona radiata |
| RightPallidum | Rightpostcentral | 0.652646 | Anterior corona radiata |
| Rightprecuneus | Leftposteriorcingulate | 0.650143 | External capsule |
| Leftposteriorcingulate | Rightprecuneus | 0.650143 | External capsule |
| Rightinsula | Leftlateraloccipital | 0.647005 | Body of corpus callosum |
| Leftlateraloccipital | Rightinsula | 0.647005 | Body of corpus callosum |
| Rightisthmuscingulate | Rightparahippocampal | 0.645539 | Cingulum (cingulate gyrus) |
| Rightparahippocampal | Rightisthmuscingulate | 0.645539 | Cingulum (cingulate gyrus) |
| Rightcaudalmiddlefrontal | RightPallidum | 0.640675 | Cerebral peduncle |
| RightPallidum | Rightcaudalmiddlefrontal | 0.640675 | Cerebral peduncle |

Garyfallidis, E., Brett, M., Correia, M.M., Williams, G.B., Nimmo-Smith, I., 2012. QuickBundles, a Method for Tractography Simplification. Front Neurosci 6, 175.

Hua, K., Zhang, J., Wakana, S., Jiang, H., Li, X., Reich, D.S., Calabresi, P.A., Pekar, J.J., van Zijl, P.C., Mori, S., 2008. Tract probability maps in stereotaxic spaces: analyses of white matter anatomy and tract-specific quantification. Neuroimage 39, 336-347.
